# Developing and implementing guidelines on culturally adapting the Addenbrooke’s cognitive examination version III (ACE-III): a qualitative illustration

**DOI:** 10.1186/s12888-020-02893-6

**Published:** 2020-10-06

**Authors:** Waquas Waheed, Nadine Mirza, Muhammed Wali Waheed, Abid Malik, Maria Panagioti

**Affiliations:** 1 Centre for Primary Care and Health Services Research, The University of Manchester, Suite 8, 5th Floor, Williamson Building Oxford Road, Manchester, M13 9PL UK; 2 The University of Leicester, Leicester, UK; 3 Greater Manchester Mental Health NHS Foundation Trust, Salford, UK

**Keywords:** Cross cultural, Ethnic minority, Non-English, Language, Psychometrics, Scale development, Transcultural, Translation

## Abstract

**Background:**

Cognitive tests currently used in healthcare and research settings do not account for bias in performance that arises due to cultural context. At present there are no universally accepted steps or minimum criteria for culturally adapting cognitive tests. We propose a methodology for developing specific guidelines to culturally adapt a specific cognitive test and used this to develop guidelines for the ACE-III. We then demonstrated their implementation by using them to produce an ACE-III Urdu for a British South Asian population.

**Methods:**

This was a several stage qualitative study. We combined information from our systematic review on the translation and cultural adaptation of the ACE-III with feedback from previous ACE-III adaptors. This identified steps for cultural adaptation. We formatted these into question-by-question guidelines. These guidelines, along with feedback from focus groups with potential users were used to develop ACE-III Urdu questions. Clinical experts reviewed these questions to finalise an ACE-III Urdu.

**Results:**

Our systematic review found 32 adaptations and we received feedback from seven adaptors to develop the guidelines. With these guidelines and two focus groups with 12 participants a sample ACE-III Urdu was developed. A consensus meeting of two psychiatrists with a South Asian background and familiarity with cognitive tests and cultural adaptation finalised the ACE-III Urdu.

**Conclusions:**

We developed a set of guidelines for culturally adapting the ACE-III that can be used by future adaptors for their own language or cultural context. We demonstrated how guidelines on cultural adaptation can be developed for any cognitive test and how they can be used to adapt it.

## Background

There is an increasing prevalence of dementia on a global scale, with numbers showing there will be a rise from 50 million to 82 million in the next decade [1]. Within the United Kingdom (UK) there was an estimated 850,000 people with dementia in 2015 but this is expected to increase to 1 million by 2021 and over 2 million by 2051 [2]. Thus, the need for early and accurate diagnosis continues to take precedence [3, 4].

In addition to a physical examination and robust interviewing [5] the diagnosis must be supplemented with the administration of a cognitive test [6]. These cognitive tests are characterised by their ability to either screen for or diagnose cognitive impairment and dementia [3]. In many cases they will also investigate severity and the potential subtype of dementia [7].

This is achieved through questions that assess individual cognitive capabilities- otherwise known as cognitive domains- such as attention and orientation, fluency, memory, language, and visuospatial abilities [5, 7]. They are often administered verbally, consisting of written, verbal, and sometimes physical tasks, ideally taking under 30 min [6].

Currently there are over 40 cognitive tests available and under use in both healthcare settings and within the context of research [8]. These range from brief screening tests such as the Six Item Cognitive Impairment Test (6 CIT) [9], Clock Drawing [10], the Mini Cog Test [11], and Test Your Memory (TYM) [12] to longer more extensive diagnostic tests such as the Mini Mental State Examination (MMSE) [13], the Montreal Cognitive Assessment (MoCA) [14], and the Addenbrooke’s Cognitive Examination Version III (ACE-III) [15].

These cognitive tests were originally designed for English speaking European countries [16], standardised on well educated, male, Caucasian outpatients [17]. While applicable to that demographic, the original versions of these cognitive tests are not suitable for use with diverse populations [18].

Within the UK alone, 88 main languages other than English are spoken [19] and 8% of the population do not have English as a first language [20]. It is also estimated that over 864,000 struggle to or are unable to speak English [20]. Cognitive tests in their current state would not be able to accommodate for these non-English speakers. Thus, many cognitive tests have been translated into targeted languages. However, these translated versions haven’t necessarily been psychometrically validated and translation alone does not address gaps in understanding and acceptability that arise due to cultural context [4].

In the UK 14% of the population identifies as belonging to an ethnic minority group [19] and this is predicted to rise to 20% by 2051 [21]. A significant proportion of these ethnic minorities migrated to the UK as young adults [22, 23]. Therefore, they still adhere in many ways to the culture of their home countries and pass this culture on to their children [22, 23].

This cultural context cannot be ignored when administering cognitive tests as culture influences how a respondent perceives test questions and how they respond to them, if they are even able to do so [18, 24]. This is due to many cognitive test questions being reliant on a familiarity with the western calendar and western names, and knowledge of western history, objects and even wildlife [4]. When these questions have not been adapted to account for cultural context bias occurs. This equates to a loss of content equivalence (the questions are not relevant to the cultural context), criterion equivalence (the questions are unable to accurately assess for dementia) and content equivalence (the questions are no longer able to accurately assess the individual cognitive domains they were designed to) [25].

This accounts for higher rates of false positive and false negative scores across cognitive tests within non –English speakers and ethnic minority groups as compared to their English speaking and Caucasian counterparts [18, 26, 27]. It also compromises the generalisability of the results of dementia research that incorporate these cognitive tests [18, 26, 27].

To counter this, attempts at designing new cognitive tests for specific groups have been tried but this was deemed too time consuming and complex, reducing feasibility [28]. Another suggestion has been to adjust cut-off scores for different ethnic minority and non-English speaking groups but this has been criticised for reducing sensitivity, specificity and likelihood ratios [29]. Therefore, culturally adapting an existing cognitive test has been regarded as a preferred alternative.

As mentioned, translating these tests does occur but this does not overcome the influence of culture beyond fluency in the target language. Existing cultural adaptations of cognitive tests do exist and have been developed through a variety of qualitative methods such as the use of global guidelines, involving experts and potential users in coproduction, and pilot testing [30]. However, at present there is no universal standard procedure or minimum criteria for culturally adapting these cognitive tests [31].

Therefore, there must be a global consensus on the steps and procedures that are essential for undertaking thorough cultural adaptation of cognitive tests [18]. These must be conducted before the adapted cognitive tests can be administered in healthcare and research settings. The final step would be a psychometric validation of the adapted cognitive test within the target population. However, prior to this there must be steps that focus on the cultural adaptation process itself.

We propose that for each cognitive test there should be a set of guidelines on how to culturally adapt the questions of that specific test. These would be developed through an incorporation of a review of previous literature on that test [25] with feedback from those who have already adapted it. These guidelines would provide step by step instructions on how to culturally adapt every question of that cognitive test in accordance with evidence to allow for the retention of content, criterion and conceptual equivalence [25].

The guidelines would then be implemented by using them to create culturally adapted versions of the cognitive test questions for a particular target demographic. These versions would be presented to potential users from the target demographic and clinical experts in the field. Their feedback would finalise which versions of the test questions will be retained in the adapted version of the cognitive test.

Following this there would be a cultural validation of the adapted cognitive test. This would consist of administering it to members of the target population and conducting qualitative cognitive interviews to assess the adapted cognitive tests understanding and acceptability within this population [32]. Once this has occurred, as mentioned, the psychometric validation would take place.

In this paper we will detail the process of developing and implementing such a set of guidelines for a non-English speaking ethnic minority group within the UK. The cognitive test we selected was the ACE-III [15], a gold standard tool for the diagnostic accuracy of cognitive impairment and dementia [33], consisting of 19 questions that assess the cognitive domains attention, memory, fluency, language and visuospatial abilities (See Table 1).

**Table 1:** Questions of the Addenbrooke’s Cognitive Examination Version III

Question Number	Task/Question
1: Attention – Orientation	Ask the day, date, month, year, season, floor, street/hospital, town, county and country.
2: Attention – Registration	Say the words lemon, key and ball and ask them to repeat and try to remember.
3. Attention – Concentration	Ask to take 7 away from 100 and keep taking 7 away from the new number for 5 trials (Serial 7’s).
4. Memory –Recall	Ask for the three words from 2. Attention – Registration.
5a. Fluency –Letters	Ask for as many words as they can think of starting with the letter ‘P’, not including names of pronouns, in one minute.
5b. Fluency –Animals	Ask for the names of as many animals as they can think of in one minute.
6. Memory –Anterograde	Say the name and address ‘Harry Barnes, 73, Orchard Close, Kingsbridge, Devon’ and ask them to repeat and try to remember.
7. Memory –Retrograde	Ask for the name of the current Prime Minister, name of the woman who was Prime Minister, name of the USA president and name of the USA president who was assassinated in the 1960s.
8. Language –Comprehension	Place a pencil and paper in front. Ask to ‘place the paper on top of the pencil’, ‘pick up the pencil but not the paper’ and ‘pass me the pencil after touching the paper’.
9. Language –Writing	Ask to write two or more complete sentences about their last holiday/weekend/Christmas, without using abbreviations.
10. Language –Repetition	Say the words caterpillar, eccentricity, unintelligible and statistician and ask them to repeat.
11. Language –Repetition	Say the proverbs ‘All that glitters is not gold’ and ‘A stitch in time saves nine’ and ask them to repeat.
12. Language –Naming	Show 12 images and ask them to name each.
13. Language –Comprehension	Ask to point to ‘the one which is associated with the monarchy’, ‘the one which is a marsupial’, ‘the one which is found in the Antarctic’ and ‘the one which has a nautical connection’ from the 12 images provided.
14. Language –Reading	Ask them to read the words sew, pint, soot, dough and height.
15a. Visuospatial Abilities - Infinity Diagram	Ask them to copy the following:
15b. Visuospatial Abilities – Wire Cube	Ask them to copy the following:
15c. Visuospatial Abilities – Clock	Ask them to draw a clock face with numbers and the hands at ten past five.
16. Visuospatial Abilities	Ask them to count the number of dots without pointing.
17. Visuospatial Abilities	Ask them to identify the fragmented letters K, M, A and T.
18. Memory –Recall	Ask for the three words from 6. Memory – Anterograde.
19. Memory – Recognition	For each word of the name and address that could not be recalled, give the options listed and ask to identify which word it was.

The ACE-III and its predecessors, the ACE [34] and ACE-Revised (ACE-R) [35] have been translated into a range of languages and incorporated into use across the globe. English versions of the ACE-III have also been adapted for the UK and the United States of America (USA). However, the ACE-III was originally designed for English speakers native to Australia, with a reliance on knowledge of the cultural background [15] and although cultural adaptation has been undertaken by adaptors to produce suitable adaptations [36–38] there are no existing standardised guidelines for the cultural adaptation of this cognitive test.

As South Asians are the UKs largest ethnic minority group, at over 6.3% of the overall population, we selected them as our target population to culturally adapt for. The language we chose to adapt in was Urdu [32], a popular South Asian language and the 4th most common language spoken in the UK [19]. Prior to this there was only one other Urdu version of the ACE-III available, which was culturally adapted for use within India [39]. Due to this it was not applicable to the cultural contexts of other South Asian countries where Urdu speakers reside [40], nor to the Urdu speaking diaspora within Canada, the Middle East, the US and the UK [41].

Though this paper only details the development and implementation process of the guidelines, the ACE-III Urdu we produced through the methods has also undergone the cultural validation process, described elsewhere [32]. This process undertook 25 cognitive interviews with cognitively healthy Urdu speaking British South Asians over the age of 60. The ACE-III Urdu will now need to undergo a psychometric validation before being made available for widespread use.

## Methods

**Fig. 1:**
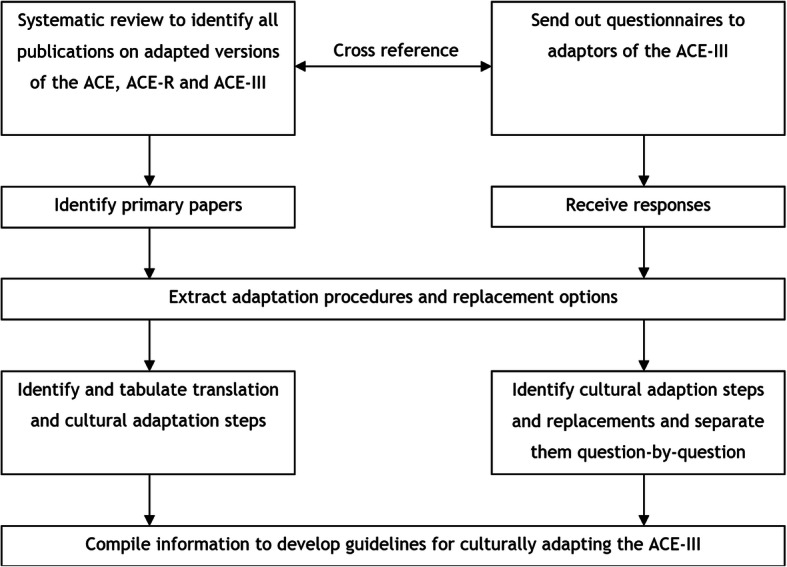
Overview of the process of developing guidelines for translating and culturally adapting the ACE-III

**Fig. 2:**
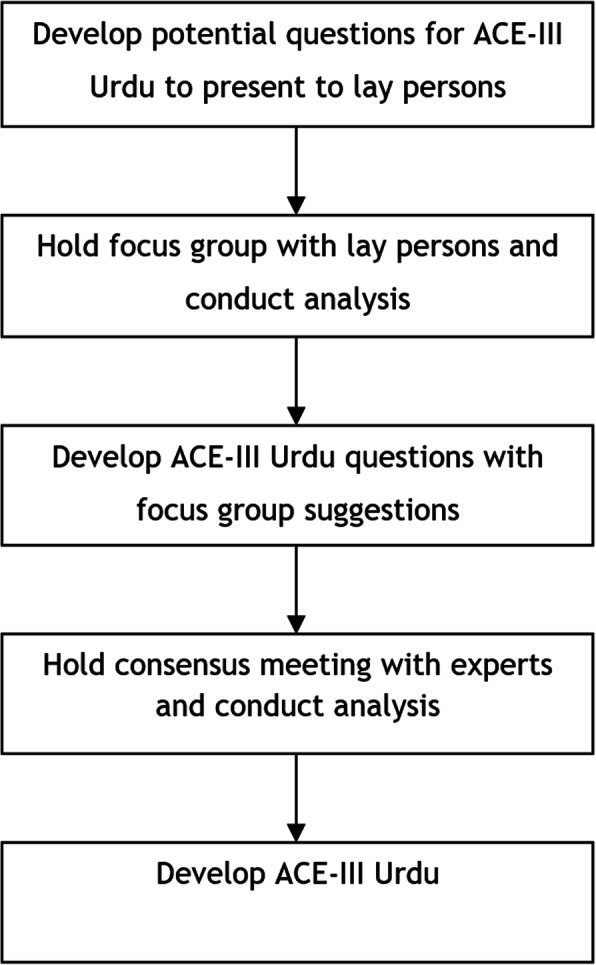
Overview of the process of utilising the guidelines to culturally adapt the ACE-III for British South Asians

A several stage qualitative approach was undertaken to develop guidelines for translating and culturally adapting the ACE-III (See Fig. 1) and implement them to develop an ACE-III Urdu (See Fig. 2):

Step 1: A systematic review.

Step 2: Receiving feedback from previous ACE-III adaptors.

Step 3: Collating the data to form guidelines.

Step 4: Implementing guidelines with feedback from lay persons and clinical experts.

Step 5: Developing the ACE-III Urdu.

### Step 1: systematic review

We conducted a systematic review of all existing primary publications of translations and cultural adaptations of the ACE-III as well as its predecessors [30].

The electronic databases EMBASE, Medline, and PsychINFO were searched using the search terms “addenbrooke’s cognitive examination or ace-iii or screen* or test or instrument or measure or tool or diagnos*”, “dementia or Alzheimer* or cognitive*” and “sensitivity and specificity or accuracy or cut-off or receiver operator or ROC or Youden” [30]. We searched from January 2013, the year the ACE-III was introduced [15], to December 2016, which was when this review was conducted. SCOPUS was also searched for any publication that cited the original paper [15]. Additionally, we also screened all included and excluded publications of a meta-analysis of the ACE and ACE-Revised (ACE-R) [42].

Publications, including validation studies, that incorporated the use of a translated or culturally adapted version of the ACE, ACE-R and ACE-III, from English into any other language, and were the primary source of that version, were included. For each of the publications we extracted data on the version of the ACE that was culturally adapted, the language it was translated into, the country it was culturally adapted for and the section of the text that described how it was culturally adapted. These reported processes were broken down into individual steps and grouped by which ACE-III question they described adapting. This allowed us to identify which questions were dependent on culture, how they had been culturally adapted, and what the rationale was behind the changes.

We also assessed the quality of the reported cultural adaptation of each publication with the Manchester Cultural Adaptation Reporting Questionnaire (MCAR) [30]. This shows which publications reported their cultural adaptation process in sufficient detail to be replicated by future adaptors.

### Step 2: feedback from adaptors of the ACE-III

We aimed to receive feedback from official adaptors of the ACE-III; those who had translated and culturally adapted it for their respective language and culture and had made their adaptation available on the Neuroscience Research Australia (NeuRA) website that hosted the original ACE-III and its implementation materials at the time of this research [39].

We downloaded all available adaptations of the ACE-III from the website. We translated them into English through the use of an online translation application and, when available, with the aid of postgraduate research students at the University of Manchester who were native speakers of the languages. The translated adaptations were read through to identify which questions in each adaptation of the ACE-III had been culturally adapted beyond a translation verbatim. For each cultural adaptation we developed a questionnaire that highlighted the questions that had been culturally adapted along with the original ACE-III counterparts (See Additional file 1 for sample questionnaire). We asked for the rationale behind changing the original question and the development process of the culturally adapted version of the question with the rationale.

We distributed the questionnaires to the corresponding adaptors attached with a standardised email relaying the purpose of the questionnaires and a request for their completion. We also requested a check of our translation of their language version. After a two week period adaptors were sent a follow up email to act as a reminder. If adaptors did not initiate any form of contact after this no further contact was made.

### Step 3: data analysis and synthesis

To develop guidelines for culturally adapting the ACE-III we collated the information from our systematic review [30] and the feedback from adaptors. We identified sets of mutually exclusive steps for culturally adapting each question of the ACE-III.

From our systematic review we had broken down the cultural adaptation processes extracted from each publication according to ACE-III question. The adaptation processes for each question across publications were merged and duplicates removed to identify the mutually exclusive steps that could be undertaken to adapt each question.

The questionnaires sent to adaptors were already organised by question. We merged the adaptors’ feedback on the cultural adaptation process of each question. We removed duplicating information so each question had mutually exclusive steps that could be undertaken to adapt that question along with the adaptors’ accompanying rationale.

The cultural adaptation steps for each ACE-III question identified from the systematic review and from the adaptors’ feedback were merged. Duplicates were removed to identify overall mutually exclusive cultural adaptation steps for each question. Accompanying rationale was presented with these steps and the respective publications and adapted versions of the ACE-III were cited, resulting in a question-by-question set of guidelines.

### Step 4: implementation of the guidelines

We conducted two focus groups within the British South Asian community of Greater Manchester. We aimed to recruit 12–14 laymen participants overall, fluent in speaking and writing Urdu, over the age of 60, able to give informed consent and who did not have a history of cognitive impairment.

Participants were voluntarily recruited via convenience sampling from the local Pakistani Community Day Centre and provided with an information sheet, available in English and Urdu. They were given 24 h to decide if they wished to participate, after which they were contacted by a liaison at the Centre to confirm their participation and let them know the date and time of the focus group. These were also held at the centre. On the day of the focus group participants would be provided with consent forms and demographics sheets, available in English and Urdu.

Using the guidelines we produced several culturally adapted versions of all questions of the ACE-III, backed up by rationale, for the British Urdu speaking population. The most suitable option would need to be selected. We presented these versions of the questions within our focus groups to receive their feedback on the questions’ cultural appropriateness, which versions should be retained for a potential ACE-III Urdu and whether they proposed any changes or suggestions of their own. This feedback was audio recorded and transcribed.

We also conducted a consensus meeting with clinical experts in the relevant fields, local to the Greater Manchester area. We aimed to recruit 2–4 experts on dementia, the cognitive testing process and the translation and cultural adaptation of these tests. They would also be familiar with the ACE-III, its rationale and how to administer it. These experts also had to be fluent in speaking and writing both English and Urdu and familiar with UK and South Asian cultures. The experts were recruited voluntarily via convenience sampling. The consensus meeting was held at the Centre for Primary Care and Health Services Research, at the University of Manchester, before which informed consent was obtained.

We presented the focus group feedback within the consensus meeting and experts determined which were the most culturally suitable adaptations of each question of the ACE-III. The consensus meeting was audio recorded, transcribed and the data was collated for each question of the ACE-III to determine how each would be culturally adapted.

### Step 5: developing the ACE-III Urdu

To develop the ACE-III Urdu the template was acquired through NeuRA, allowing the ACE-III Urdu to retain the exact same format as the original ACE-III. Urdu is read from right to left so the template was reversed horizontally such that questions were presented on the right side of the template and the scoring instructions on the left side. Standard information requested prior to the administration of the ACE-III and instructions for the implementation of the questions were translated into Urdu and typed out. Each ACE-III question was typed out and designed according to the suggestions confirmed within the consensus meeting.

## Results

### Step 1: systematic review

We identified 113 publications through our search, of which 32 met out criteria for data extraction and analysis; 12 for the original ACE, 17 for the ACE Revised and 3 for the ACE-III. Overall, these publications spanned 18 languages; Arabic, Cantonese, Chinese, Czech, Danish, French, German, Greek, Hebrew, Italian, Japanese, Korean, Lithuanian, Malayalam, Persian, Portuguese, Slovak and Spanish [30].

The full results of the systematic review are described elsewhere [30].

### Step 2: feedback from adaptors of the ACE-III

Our search of the NeuRA website identified 17 fully adapted versions of the ACE-III for the languages Egyptian Arabic, Saudi Arabian Arabic, Chinese, Estonian, Hebrew, Hindi, Hungarian, Indian Kannada, Italian, Japanese, Marathi, Polish, Portuguese, Spanish, Tamil, Telugu and Indian Urdu. Three versions had been retained in English but culturally adapted for India, New Zealand and the USA, resulting in a total of 20 ACE-III adaptations.

Of these adaptations the Estonian, Indian Kannada, Marathi, Japanese, Tamil and Telugu versions could not be translated into English due to a lack of resources in terms of translation applications and translators. They excluded from our analysis (30%). Questionnaires were developed for the remaining 14 adaptations (70%) and distributed to their respective adaptors, of which a total of seven questionnaires (35%) were returned to us fully completed.

The original Australian ACE-III was used by the Hindi, Hungarian and Spanish adaptors (15%) and the UK version of the ACE-III was used by the Egyptian Arabic, Hebrew and Welsh adaptors (15%) for their own adaptations. Polish adaptors used both (5%).

Table 2 summarises which questions of the ACE-III were culturally adapted by which adaptors, thereby showing the frequency of reported cultural adaptation undertaken for each question. Table 3 shows examples of the culturally adapted versions of these questions across a range of languages and cultural contexts. We can see that all adaptors culturally adapted questions 6, 7, 18 and 19 for memory and questions 10, 11 and 14 for language, and the majority had adapted question 2 for attention. In contrast, none of the adaptors had culturally adapted any of the questions assessing visuospatial abilities. This highlights which cognitive domains, and their respective questions, rely on culture and which would suffice with a simple translation into the target language.

**Table 2:** Questions of the ACE-III that were culturally adapted by the adaptors

ACE-III Questions																						
Language of the adaptors	1	2	3	4	5a	5b	6	7	8	9	10	11	12	13	14	15a	15b	15c	16	17	18	19
Egyptian Arabic					x		x	x			x	x	x	x	x					x	x	x
Hebrew		x			x		x	x			x	x			x						x	x
Hindi					x		x	x			x	x	x	x	x					x	x	x
Hungarian							x	x			x	x			x						x	x
Polish		x			x		x	x			x	x	x	x	x						x	x
Spanish		x					x	x			x	x		x	x						x x	x
Welsh		x			x		x	x			x	x			x						x	x

x = cultural adaptation was undertaken

**Table 3:** Examples of cultural adaptation of adapted ACE-III questions

ACE-III Question	Example of adapted version	Language of version
2: Attention – Registration: Say the words lemon, ask them to repeat and try to remember.	Lemon was changed to Sliwka (Plum).	Polish
5a. Fluency –Letters: Ask for as many words as they can think of starting with the letter ‘P’, not including names of pronouns, in one minute.	‘P’ sound was changed to ‘Sh’ sound.	Egyptian Arabic
6. Memory –Anterograde: Say the name and address ‘Harry Barnes, 73, Orchard Close, Kingsbridge, Devon’ and ask them to repeat and try to remember.	The name and address were changed to ‘Katona Péter, Tavasz utca 42., Gyöngyös, Heves megye’.	Hungarian
7. Memory –Retrograde: Ask for the name of the current Prime Minister.	Asked for the name of the current President.	Spanish
10. Language –Repetition: Say the word caterpillar.	Caterpillar was changed to Colomennod (Pigeons).	Welsh
11. Language –Repetition: Say the proverb ‘A stitch in time saves nine’ and ask them to repeat.	The proverb was changed to ‘The orchestra played and the audience applauded’	Hebrew
12. Language –Naming: Show image of kangaroo and ask them to name each.	The image was changed to a goat.	Hindi
13. Language –Comprehension: Ask to point to ‘the one which is a marsupial’, from the 12 images provided.	The question was changed to point to ‘the one which flies’.	Egyptian Arabic
14. Language –Reading: Ask them to read the word height.	Height was changed to Zamarzniety (Frozen).	Welsh
17. Visuospatial Abilities: Ask them to identify the fragmented English letters.	The letters were changed to Hindi alphabet.	Hindi

### Step 3: data analysis and synthesis

**Fig. 3:**
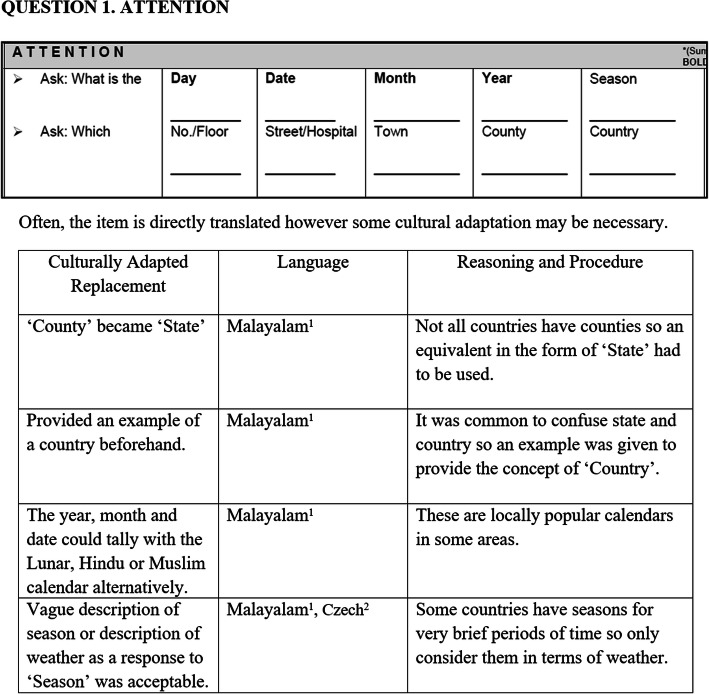
An example page of the guidelines

For each question of the ACE-III, the individual cultural adaptation steps identified from our systematic review and from adaptors’ feedback, along with rationale undertaken, were tabulated to form the guidelines (See example in Fig. 3 and see Addtional file 2). For each question the following was presented:

1. How the question has been previously culturally adapted with the steps undertaken.
2. Examples compiled from publications and the questionnaires, citing the respective languages and adaptors of the ACE-III
3. The rationale behind adapting the question and choosing the adapted replacement.

### Step 4: implementation of the guidelines

Our focus groups had 12 voluntary participants, five female (41%) and seven male (59%), from ages 61–75 years (M = 66.67, SD = 6.44), from the Greater Manchester area. (See Table 4 for a breakdown of participant demographics). Seven participants were married, three were widowed, and two did not disclose. Six were retired, three were housewives, two were unemployed, and one did not disclose. Participants came from varied socio-economic backgrounds, across a range of educational backgrounds.

**Table 4:** Demographic details of focus group participants

PI	Age	Level of Education	First Language	Other Language
1F	60s	X	Punjabi	Urdu, English
2F	70s	10th Year	Punjabi	Urdu
3F	60s	FA	Punjabi	Urdu
4F	60s	FA	Punjabi	Urdu
5F	70s	None	Punjabi	Urdu
1 M	70s	Graduate	Punjabi	Urdu, English
2 M	60s	BA	Urdu	English
3 M	70s	MA	Punjabi	Urdu, English, Arabic
4 M	70s	GCSEs	Urdu	English
5 M	60s	GCSEs	Urdu	Punjabi, English
6 M	60s	X Graduate	Punjabi	Urdu, Italian, English
7 M	70s	Graduate	Punjabi	Urdu, English, Persian

Through our proposed suggestions for each question of the ACE-III Urdu, developed through the use of the guidelines, we determined questions 5b, 8, 9, 15a, 15b, 15c and 16 would suffice with a direct translation. The remaining questions required further cultural adaptation that were deliberated over during these focus groups (See Additional file 3 for the proposed suggestions, developed using the guidelines).

Our consensus meeting was attended by two experienced old age psychiatrists who were both bilingual British Pakistanis. They had lived in both the UK and Pakistan and were familiar with the cultures of both countries. They were also both involved in clinical and research work relevant to South Asian populations and were knowledgeable about cognitive assessments, the ACE-III and the translation and cultural adaptation of cognitive tests.

### Step 5: developing the ACE-III Urdu

The suggestions finalised within this consensus meeting and incorporated to form the ACE-III Urdu [19] can be seen in Table 5.

**Table 5:** ACE-III Urdu items determined from focus groups and consensus meeting

Suggestions	Justification
**1: Attention**	
i. Ask the question ‘These days which of the four seasons is it?’ ii. ‘Hospital’ and ‘county’ will be spelt using Urdu letters. iii. Only accept dates in the English calendar.	Participants and experts agreed with the proposed suggestions developed with rationale from the guidelines.
**2: Attention**	
i. ‘Lemon’ is directly translated into Urdu. ii. ‘Key’ is replaced with ‘bell’, spelt using Urdu letters. iii. ‘Ball’ is directly translated into Urdu	Participants and experts agreed with the proposed suggestions developed with rationale from the guidelines for i and ii. For iii, participants said ‘bell’ and ‘ball’ sound far too alike and it was decided that ‘ball will be directly translated as the Urdu word for ‘ball’ is also one syllable.
**3: Attention**	
Use the word ‘minus’, spelt using Urdu letters and the Urdu translation for ‘take away’.	Participants and experts agreed with the proposed suggestions developed with rationale from the guidelines.
**4: Memory**	
Refer to Question 2: Attention	Refer to Question 2: Attention
**5a: Fluency (Letters)**	
Replace the letter ‘P’ with the Urdu letter چ (chay).	Participants agreed with both proposed suggestions developed with rationale from the guidelines. Between the letters چ (chay) and گ (gaaf), the former was then chosen by experts after debate due to its unique sound as the latter could be mistaken for the similar sounding letter ک (kaaf).
**6: Memory**	
i. The first name Haroon is used. The last name Butt is used. ii. The original ACE-III address will be retained and spelt using Urdu letters.	Participants agreed with all the proposed suggestions developed with rationale from the guidelines. The name Haroon Butt was settled upon by the experts due to it retaining the sounds and length of the original name, Harry Barnes.
**7: Memory**	
i. The first, second and third question of the original ACE-III, ‘Name of the current Prime Minister’, ‘Name of the first female Prime Minister’ and ‘Name of the USA president’ are retained. ii. The fourth question will be replaced with ‘Name of the princess who died in a car crash in the 1990s’.	Participants agreed with the proposed suggestions developed with rationale from the guidelines. The second question was retained according to the guidelines, despite what was said in the focus groups. Experts ruled that British Urdu speaking elderly should be aware of the first female Prime Minister of the UK due to her prominence. She would have been Prime Minister at the time when many of the elderly would have initially immigrated to the UK and would therefore know of her. The fourth question was replaced with a new suggestion by the experts. Despite following the rationale of the guidelines ‘Name of the British currency’ and ‘Name of the city where (a ‘Wonder of the World’) is located’ were deemed too easy. It was also agreed that these replacements do not retain the conceptual equivalence of the question. ‘Name of the princess who died in a car crash in the 1990’ retains the concept, relating to a well know historical death relevant to the UK.
**10: Language**	
i. ‘Eccentricity’ is replaced with گوش گزار ii. ‘Unintelligible’ is replaced with نشیب و فراز iii. The Indian Urdu replacements for ‘caterpillar’ and ‘statistician’ are retained.	Participants agreed with the proposed suggestions developed with rationale from the guidelines. ‘Eccentricity’ was replaced with a new suggestion by experts as the words proposed were deemed too easy in comparison to the original counterparts.
**11: Language**	
The first saying, ‘All that glitters is not gold’, is translated into Urdu. The second saying is replaced with the saying that translated to ‘You cannot clap with one hand’.	Participants and experts agreed with the proposed suggestions developed with rationale from the guidelines.
**12: Language**	
i. Spoon is retained. ii. Book is retained. iii. Kangaroo is replaced with a goat. iv. Penguin is replaced with a peacock. v. Anchor is replaced with scissors. vi. Camel is retained. vii. Harp is replaced with a dohl. viii. Rhino is replaced with a bear. ix. Barrel is replaced with a suitcase. x. Crown is replaced with a cap. xi. Crocodile is replaced with a tortoise. xii. Accordion is replaced with a trumpet.	Participants agreed with the proposed suggestions developed with rationale from the guidelines. For iii, experts decided that though sheep are more common in the UK, British Urdu speakers would be familiar and able to recognise a goat. For iv, experts selected a peacock as British Urdu speakers would be more familiar with it as opposed to a parrot. For v, experts settled on scissors as they are a common household object. For vii, experts selected a dohl due to familiarity with it in the culture. For viii, experts proposed the new suggestion of a bear to replace the rhino as it is a better known wild animal but still unique in the UK. Experts ruled that a lion could be confused with other big cats such as a tiger and a monkey is not as relative to the cultural context of the UK. For ix, experts selected a suitcase as it is a form of container with a specific purpose. For x, experts proposed the new suggestion of a cap as it is a better known form of headwear. For xi, experts proposed the new suggestion of a tortoise, because it is a better known wild animal that would be better recognised by the British Urdu speaking elderly. For xii, a trumpet was selected out of the proposed instruments by participants from the focus groups as it was considered the most uniquely shaped and easily recognisable by British Urdu speaking elderly.
**13: Language**	
The following questions were asked regarding the images: ‘Which one is related to the head’, ‘Which one is found in the desert’, ‘Which one has a shell on it’ and ‘Which one is related to travel’.	All questions were developed by the authors NM and WW according to the images that were finalised, following the guidelines. Participants and experts agreed with the proposed questions.
**14: Language**	
The words used in the Indian Urdu ACE-III were retained.	Participants and experts agreed with the proposed suggestions developed with rationale from the guidelines
**17: Visuospatial Abilities**	
The letters و، م،ی،ا were selected.	Participants and experts agreed with the proposed suggestions developed with rationale from the guidelines
**18: Memory**	
Refer to Question 6: Memory.	Refer to Question 6: Memory.
**19: Memory**	
Refer to Question 6: Memory. The names Jamal Butt and Haroon Khan replaced the original names for recognition.	Refer to Question 6: Memory. Participants agreed with the proposed suggestions developed with rationale from the guidelines. The alternative names chosen by the experts retained the length, familiarity and number of syllables.

## Discussion

In this paper we detailed the methods involved in developing a set of guidelines to culturally adapt a specific cognitive test; in this instance, the ACE-III. Following this we also demonstrated how these guidelines can be implemented to develop a cultural adaptation of the test it was designed for, for a specific target population.

The combination of the systematic review and adaptors’ feedback provided us with detailed information on the cultural adaptation of the ACE-III, which was used to develop question-by-question guidelines. These present culturally adapted versions of questions of the ACE-III, backed up by rationale.

We could potentially have incorporated more cultures and languages through translators but due to limited resources we were unable to produce questionnaires for 6 of the existing adaptations. We also had a poor return rate of questionnaires (35%), reducing the amount of additional information we could have received and compiled into the guidelines. Since our review and this research more cultural adaptations of the ACE-III have also been released that could not be included at this time.

Therefore we must acknowledge that the usefulness of these guidelines, and any guidelines for cognitive tests developed via these methods, is dependent on how many language and cultural versions of that test have been developed and how accessible these versions are. They are also limited by how many current adaptors of these versions provide rich data on the rationale behind culturally adapting questions that is often not conveyed through publications alone.

However, the methods for forming these guidelines are designed to allow for necessary updates as more information on cultural adaptation is acquired. In this way newer additions can be made by simply adding in any additional cultural adaptation steps found from new publications on adaptations of the ACE-III. The inclusion of feedback from adaptors also ensures we are not limited strictly to just published information.

In addition, we were still able to account for 22 international languages and cultural contexts in our guidelines. Through the frequency of cultural adaptation across questions, evidenced in our systematic review [30] and from the feedback of the adaptors, we were also able to determine which questions would most likely require cultural adaptation and which could suffice with a simple translation. The guidelines highlight this. With the accompanying rationale these guidelines would allow future adaptors to conduct their own cultural adaptation of the ACE-III and we have demonstrated this through our cultural adaptation of the ACE-III for a British Urdu speaking population.

While utilising the guidelines to develop potential questions for an ACE-III Urdu [32] we acknowledged British South Asians’ preferences for certain English words that are spelt with Urdu letters, such as, ‘county’, ‘bell’ and ‘ball’, as opposed to translating words into Urdu. This is attributed to the mixing of English and Urdu that occurs within British Urdu speaking communities. We also noted the influence of the structure in which sentences are presented in Urdu, and proposed the rephrasing of questions to avoid confusion. This can be seen with the elaboration of ‘What is the season?’ to ‘Which of the four seasons is it?’ due to the Urdu word for weather and season being the same.

We presented these suggestions within our focus groups, allowing us to gather feedback from men and women of a vast array of educational backgrounds within the British Urdu speaking community. Throughout the discussion participants were able to follow the rationale provided by the guidelines when proposing suggestions for the ACE-III Urdu. There was a notable insistence on cultural adaptation for questions assessing memory and language, with little focus on fluency and visuospatial abilities. This is not to say that questions of fluency and visuospatial abilities may not require cultural adaptation, only that for this target population they did not.

Following this we conducted a consensus meeting with experts to review suggestions for the ACE-III Urdu proposed during the focus groups. There was a general consensus with the suggestions proposed, barring a few items. Within ‘Question 2: Attention’ experts preferred ‘ball’ be translated directly into Urdu and in the case of ‘Question 7: Memory – Retrograde’ experts decided to retain three questions as they were. The proposed suggestions were deemed too easy and the original questions were at a specific level of difficulty that was required to measure retrograde memory.

Overall this demonstrated the usability of the guidelines and their role in developing cultural adaptations of cognitive test questions alongside both potential user and clinical expert feedback. These methods also allowed us to develop a familiarity for the cognitive test we were working with, and the intricacies of the various test questions, in a way that general guidelines do not allow.

Through our methods we also developed the first version of the ACE-III Urdu that can be used in the cultural context of the UK. As mentioned earlier the next step was to conduct a cultural validation, which assessed the ACE-III Urdu’s understanding and acceptability across older Urdu speaking British South Asians [32]. Further efforts will now be undertaken to determine its performance in the detection of dementia through a psychometric validation.

Overall, these methods can be replicated for any cognitive test to develop guidelines for adaptation specific to that test. Similarly, they can also be implemented in the same manner for any target population.

## Conclusions

The guidelines are the first of their kind, and we have provided an in depth account of the approach we undertook to develop them and implement them. This was not restricted to published literature but incorporated the first hand experiences of cultural adaptation by existing adaptors of the ACE-III. This accounted for adaptations that may not have had corresponding publications. Furthermore, instead of adhering to general guidelines on cultural adaptation, developing guidelines designed for the cultural adaptation of the ACE-III allowed for familiarity with its individual questions.

These guidelines for the ACE-III can now be implemented in the same manner we conducted for other language and cultural groups, with the added incorporation of potential user and clinical expert feedback. The implications of this methodology can also be taken forward to develop guidelines in the same manner for other existing cognitive tests. This would help to alleviate issues with mismatched culture across diverse populations that we currently see within healthcare and research settings.

## Supplementary information

**Additional file 1: Supplementary Material- Appendix A1.** Sample Questionnaire- “Cultural adaptation process of the Italian ACE-III”.

**Additional file 2: Supplementary Material- Appendix A2.** The first edition of our guidelines on translating and culturally adapting the ACE-III.

**Additional file 3: Supplementary Material- Appendix A3.** Table on ‘Proposed ACE-III questions developed from the guidelines’.

## Data Availability

Not Applicable.

## Abbreviations

UK: United Kingdom
6 CIT: six item cognitive impairment test
TYM: Test Your Memory
MMSE: Mini Mental State Examination
MoCA: Montreal Cognitive Aessment
ACE-III: Addenbrooke’s Cognitive Examination Version III
USA: United States of America
ACE-R: ACE Revised
MCAR: Manchester Cultural Adaptation Reporting Questionnaire
NeuRA: Neuroscience Research Australia
