# Supplementary material for: Developing and implementing guidelines on culturally adapting the Addenbrooke’s cognitive examination version III (ACE-III): a qualitative illustration
Source: BMC Psychiatry. 2020 Oct 6;20:492. doi: 10.1186/s12888-020-02893-6 (PMC7539399; doi:10.1186/s12888-020-02893-6)
Supplement: Supplementary file 1 — Additional file 1: Supplementary Material- Appendix A1. Sample Questionnaire- “Cultural adaptation process of the Italian ACE-III”. [file 12888_2020_2893_MOESM1_ESM.doc]

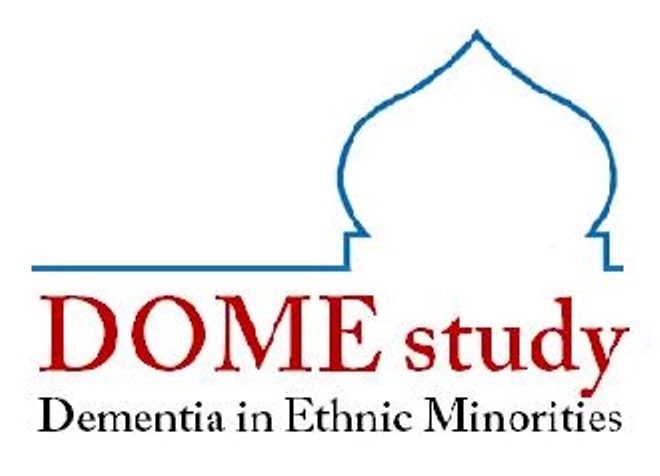


**CULTURAL ADAPTATION PROCESS OF THE ITALIAN ACE-III**

You have been asked to complete the following questionnaire which will ask you to elaborate on the adaptation process you undertook when developing the Italian Addenbrooke’s Cognitive Examination Version III. Questions will refer to items that were changed. Upon completion please return to Nadine Mirza at nadine.mirza@postgrad.manchester.ac.uk

Q1: Which version of the ACE-III did you adapt from? eg. The Australian ACE-III.

Answer:

Q2: Was the Italian ACE-III mentioned in any publications? If so please list.

Answer:

Q3a: In reference to the second “Attention”, what was the justification for changing the words?

ITALIAN:

- casa
- pane
- gatto

AUSTRALIAN:

- lemon
- key
- ball

Answer:

Q3b: How did you select the words that would take their place? What was the rationale behind this selection?

Answer:

Q4a: In reference to the first “Fluency” item, what was the justification for changing the letter?

AUSTRALIAN:

The letter I want you to use is the letter “P”

ITALIAN:

| Ha un minuto di tempo e la lettera è “F” |
| --- |

Answer:

Q4b: How did you select the letter that would take its place? What was the rationale behind this selection?

Answer:

Q5a: In reference to the second “Memory” item, what was the justification for changing the name and address?

AUSTRALIAN:

Harry Barnes

73 Market Street

Rockhampton

Queensland

ITALIAN:

Mario Rossetti

Piazza Garibaldi 59

Pontedera

Pisa

Answer:

Q5b: How did you select the address that would take its place? What was the rationale behind this selection?

Answer:

Q6a: In reference to the third “Memory” item, what was the justification for making changes to the questions?

ITALIAN:

- Nome dell’attuale Presidente della Repubblica?
- Nome del precedente Papa?
- Nome del presidente degli USA
- Nome del presidente degli USA assassinato negli anni Sessanta ?

AUSTRALIAN:

- Name of the current Prime Minister?
- Name of the Premier of New South Wales?
- Name of the USA president?
- Name of the USA President who was assassinated in the 1960s?

Answer:

Q6b: How did you select the questions that would take its place? What was the rationale behind this selection?

Answer:

Q7a: In reference to the third “Language” item, what was the justification for changing the words?

AUSTRALIAN:

- caterpillar
- eccentricity
- unintelligible
- statistician

ITALIAN:

- pagliaccio
- deformazione
- irresponsabilità
- slittino

Answer:

Q7b: How did you select the words that would take their place? What was the rationale behind this selection?

Answer:

Q8a: In reference to the fourth “Language” item, what was the justification for changing the phrases?

AUSTRALIAN:

- All that glitters is not gold.
- A stitch in time saves nine.

ITALIAN:

- L’abito non fa il Monaco.
- Chi fa da se fa per tre

Answer:

Q8b: How did you select the phrases that would take their place? What was the rationale behind this selection?

Answer:

Q9a: In reference to the seventh “Language” item, what was the justification for changing the words?

AUSTRALIAN:

 sew

 pint

 soot

 dough

 height

ITALIAN:

- ruvido
- cucciolo
- includere
- minimo
- orfano

Answer:

Q9b: How did you select the words that would take their place? What was the rationale behind this selection?

Answer:

Q10a: In reference to the fifth “Visuospatial Abilities” item what was your justification for changing the first letter?

AUSTRALIAN:


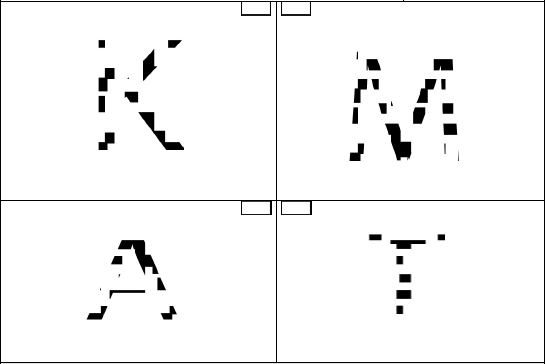


ITALIAN:


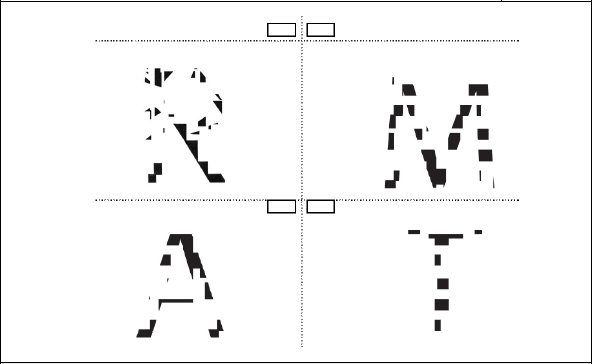


Answer:

Q10b: How did you select the letters (sounds) that would take their place? What was the rationale behind this selection?

Answer:

Q11a: In reference to the last “Memory – Recall” item, what was the justification for changing the name and address options?

AUSTRALIAN:

Jerry Barnes

37

Market Road

Townsville

Queensland

Harry Barnes

73

Martin Street

Rockhampton

New South Wales

Harry Bradford

76

Market Street

Cairns

Victoria

ITALIAN:

Mauro Rossi

Piazza Garibaldi

39

Ponsacco

Lucca

Mario Rossetti

Piazza Galimberti

52

Pontedera

Pistoia

Mario Rosati

Via Garibaldi

59

Empoli

Pisa

Answer:

Q11b: How did you select the names and addresses that would take their place? What was the rationale behind this selection?

Answer:

**Thank you for completing this questionnaire.**
