# Supplementary material for: Developing and implementing guidelines on culturally adapting the Addenbrooke’s cognitive examination version III (ACE-III): a qualitative illustration
Source: BMC Psychiatry. 2020 Oct 6;20:492. doi: 10.1186/s12888-020-02893-6 (PMC7539399; doi:10.1186/s12888-020-02893-6)
Supplement: Supplementary file 2 — Additional file 2: Supplementary Material- Appendix A2. The first edition of our guidelines on translating and culturally adapting the ACE-III. [file 12888_2020_2893_MOESM2_ESM.doc]

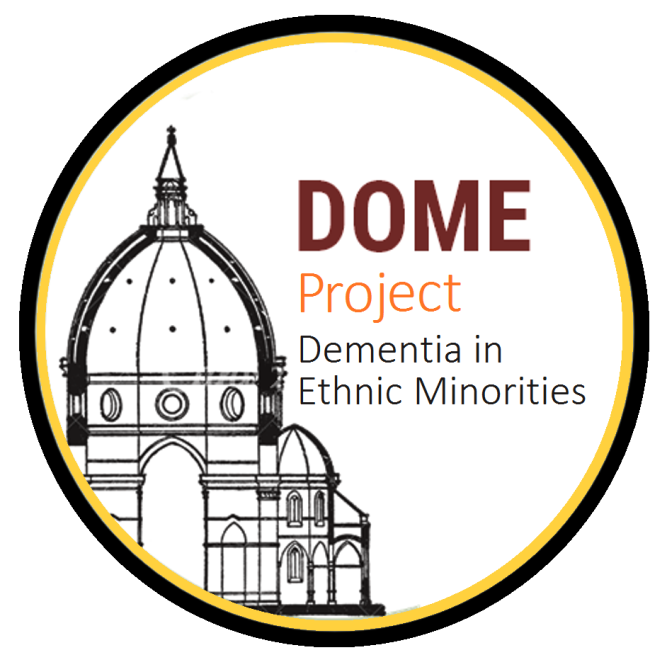
Appendix A1

**Guidelines on Translating and Culturally Adapting**

The Addenbrooke’s Cognitive Examination Version III (ACE-III)

**VERSION I**

Developed by

**Nadine Mirza** and **Dr. Waquas Waheed**

Centre for Primary Care

The University of Manchester

**(2016)**

CONTENTS

| **Acknowledgements & Copyright** | |  | **2** |
| --- | --- | --- | --- |
| **Translation Steps** |  | | **3** |
| **Cultural Adaptation** |  | | **4** |
| **Item 1: Attention** |  | | **5** |
| **Item 2: Attention** |  | | **6** |
| **Item 3: Attention** |  | | **7** |
| **Item 4: Memory** |  | | **8** |
| **Item 5: Fluency (Letters)** |  | | **9** |
| **Item 5: Fluency (Animals)** |  | | **11** |
| **Item 6: Memory** |  | | **12** |
| **Item 7: Memory** |  | | **15** |
| **Item 8: Language** |  | | **19** |
| **Item 9: Language** |  | | **20** |
| **Item 10: Language** |  | | **21** |
| **Item 11: Language** |  | | **23** |
| **Item 12: Language** |  | | **25** |
| **Item 13: Language** |  | | **29** |
| **Item 14: Language** |  | | **31** |
| **Item 15: Infinity Diagram** |  | | **34** |
| **Item 15: Wire Cube** |  | | **35** |
| **Item 15: Clock** |  | | **36** |
| **Item 16: Visuospatial Abilities** |  | | **37** |
| **Item 17: Visuospatial Abilities** |  | | **38** |
| **Item 18: Memory** |  | | **40** |
| **Item 19: Memory** |  | | **41** |

**References 42**

**ACKNOWLEDGEMENTS**

Many thanks to *FRONTIER*, the frontotemporal dementia clinical research group at the University of Sydney, for providing the Addenbrooke’s Cognitive Examination and its revisions and contact information for the adaptors of this assessment and to *Dr. A.J. Larner* for assisting in identifying the publications we reviewed for these guidelines.

I would also like to thank *Dr. J.P Newman*, *Dr. Norbert Kovacs*, *Dr. Emilia Sitek*, *Dr. Jordi Matias-Guiu Antem*, *Dr. Suvarna Alladi* and *Dr. Shailaja Mekala*, *Prof. Gwerfyl Roberts*, and *Dr. Tarik Qassem* for their responses to questionnaires that enquired about the cultural adaptation process they undertook which informed these guidelines.

**COPYRIGHT**

*Prof. John Hodges* is the copyright holder of both the ACE-R and the ACE-III. Any proposed changes or translations developed, including those aided by these guidelines, must be submitted for approval to *FRONTIER*, at the University of Sydney prior to implementation (frontier@sydney.edu.au).

For further information regarding these guidelines please contact *Nadine Mirza* (nadine.k.mirza@gmail.com).

**TRANSLATION STEPS**

The items of the ACE-III will require translation into a target language. The following summarises the translation steps that have been undertaken by previous adaptors of the ACE, ACE-R and ACE-III.

1. Translation: Direct translation, without any form of cultural adaptation, from English into the target language, often with the assistance of a native or fluent speaker of the language or an official translator.
2. Back Translation: Creating a retroversion of the initial translation, from the target language back to English, often with the assistance of a native or fluent speaker of the language or an official translator.
3. Users in Coproduction: Potential or future users of the assessment, including native and fluent speakers of the language, providing feedback or information in any way that influences the development of the translated assessment.
4. Expert Recommendations: Experts on translation, the target languages, or subject matters related to the assessment providing feedback or information in any way that influences the development of the translated assessment.
5. Revisions based on step-by-step feedback: Constant and continuous revisions of the translated assessment informed by feedback as soon as it is presented.
6. Involvement of the original authors: Authors of the original assessment providing feedback or information in any way that influences the development of the translated assessment.
7. Pilot Study: Administering translated versions of the assessment.

**CULTURAL ADAPTATION**

The items of the Addenbrooke’s Cognitive Examination that require cultural adaptation are identified in these guidelines. These guidelines detail how each item has been previously culturally adapted across several languages in the Addenbrooke’s Cognitive Examination (ACE), Addenbrooke’s Cognitive Examination Revised (ACE-R) and Addenbrooke’s Cognitive Examination Version III (ACE-III). Following the reasoning and procedures detailed below, an item-by-item cultural adaptation of the ACE-III can be conducted.

**ITEM 1. ATTENTION**

**
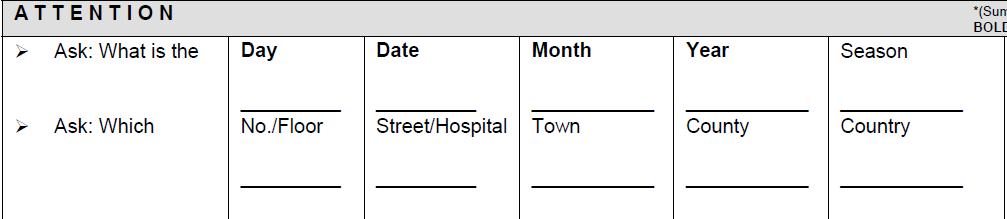
**

Often, the item is directly translated however some cultural adaptation may be necessary.

| Culturally Adapted Replacement | Language | Reasoning and Procedure |
| --- | --- | --- |
| ‘County’ became ‘State’ | Malayalam^1^ | Not all countries have counties so an equivalent in the form of ‘State’ had to be used. |
| Provided an example of a country beforehand. | Malayalam^1^ | It was common to confuse state and country so an example was given to provide the concept of ‘Country’. |
| The year, month and date could tally with the Lunar, Hindu or Muslim calendar alternatively. | Malayalam^1^ | These are locally popular calendars in some areas. |
| Vague description of season or description of weather as a response to ‘Season’ was acceptable. | Malayalam^1^, Czech^2^ | Some countries have seasons for very brief periods of time so only consider them in terms of weather. |

**ITEM 2. ATTENTION**

**
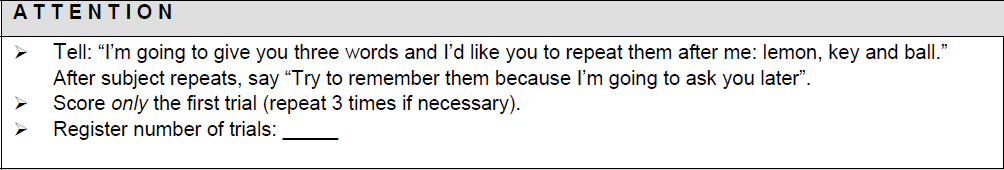
**

Often, the item is directly translated however some cultural adaptation may be necessary.

| Culturally Adapted Replacement | Language | Reasoning and Procedure |
| --- | --- | --- |
| ‘Key’ was changed to ‘Train’ or ‘Bell’. | Castillian Spanish^3^, Spanish, Welsh | The length, frequency and imaginability of the item were retained.  ‘Key’ is ‘Llave’ in Castillian Spanish, which is two syllables. ‘Train’ is ‘Tren’ in Spanish and is only one syllable, retaining the length.  ‘Key’ is ‘Goriad’ in Welsh, which is two syllables. As there was no relationship between the words the substitute ‘Cloch’ was chosen, a one syllable word that means ‘Bell’. This was also chosen because like ‘Key’, ‘Bell’ is related to accessing a door. |
| ‘Lemon’ was replaced with ‘Orange’ or ‘Plum’. | Chinese^4^, Polish | Replacement items had to be the appropriate length, imaginability and frequency as the originals.  ‘Lemon’ is ‘Cytryna’ in Polish, which is three syllables. ‘Plum’ is ‘Sliwka’ in Polish and is two syllables. |

**ITEM 3. ATTENTION**

**
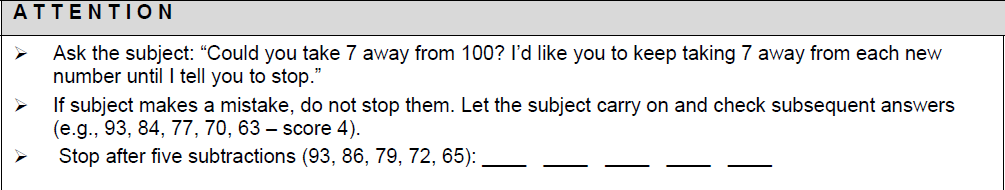
**

This item was translated directly and required no cultural adaptation.

**ITEM 4. MEMORY**

**
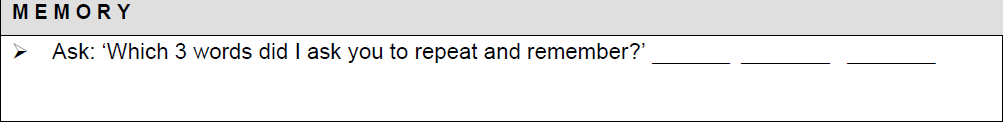
**

Refer to Item 2: Attention.

**ITEM 5. FLUENCY (Letters)**

**
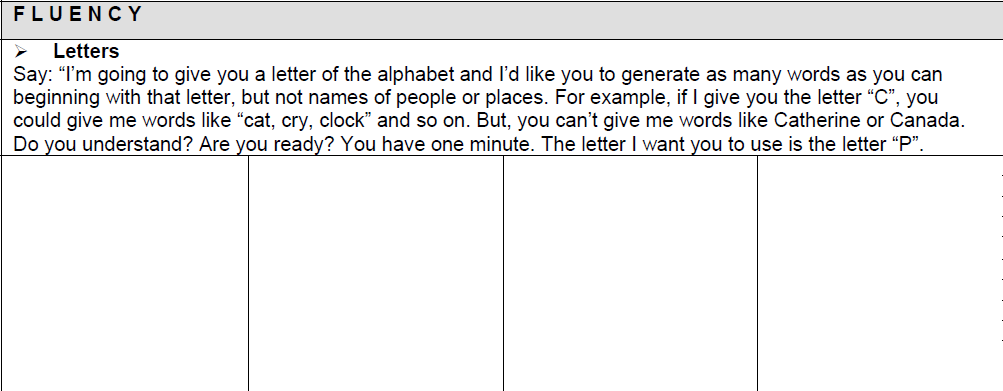
**

Instructions for this item were translated directly.

| Culturally Adapted Replacement | Language | Reasoning and Procedure |
| --- | --- | --- |
| ‘P’ was replaced with the syllable ‘ka’ or ‘sa’. | Japanese^5,6,7^ | The Japanese language is based on syllables. The method of scoring was also changed because the Japanese phonological fluency test might be more difficult than English letter fluency test.* |
| ‘P’ was replaced with the character ‘che’. | Chinese^4^ | The Chinese language is based on characters. The character ‘che’ also retained verbal fluency. |
| ‘P was replaced with ‘F’ ‘K’. | Italian^8^, Polish | The letter ‘F’ is commonly used in Italian fluency tests.  The letter ‘K’ is commonly used in Polish fluency tests. |
| ‘P’ was replaced with ‘S’. | Korean^9^ | The letter ‘S’ is commonly used in Korean fluency tests.  Also, words that start with the letters ‘P’ are rare in Korean. |
| ‘P’ was replaced with ‘B’. | Welsh, Persian^10^ | Words that start with ‘P’ are more common in Welsh than English. The letter ‘B’ retains the same frequency as the letter ‘P’ in English.  The letter ‘B’ was identified as the suitable equivalent to the letter ‘P’ in Persian after running a pilot study with the letters ‘B’, ‘P’ and ‘D’. |
| ‘P’ was replaced with ‘م’ (meem). | Saudi Arabian Arabic^11^ | Words that start with ‘م’ are very common in Arabic, with low level of difficulty. |
| ‘P’ was replaced with ‘प’ (pa). | Hindi | The letter ‘प’ is commonly used in Hindi fluency tests. It is equivalent to the letter ‘P’ and has the same phonetic sound.  Also, words that start with the letter ‘प’ are common in Hindi. |
| ‘P’ was replaced with ‘Sheen’ and ‘**מ’** | Egyptian Arabic, Hebrew | There is no equivalent to the letter ‘P’ in Egyptian Arabic.  The letter ‘Sheen’ has an average frequency and its pronunciation is the same across various dialects.  Also, the letter ‘Sheen’ is unaffected by a lisp.  There is no letter ‘P’ in Hebrew. Words that start with the letter ‘**מ**’ are some of the most common in Hebrew. |
| The task of naming as many vegetables as possible in one minute. | Cantonese Chinese^12^ | There is no equivalent letter fluency task in Cantonese. A category fluency task was chosen instead. |

*The mean scores of letter fluency for the initial letter “P” were 11.3–13.9 words for cognitively normal elderly (65–74 year olds) in an urban US community (Ganguli et al.,2010). Meanwhile, the mean scores of phonological fluency for the initial syllable “ka” were 7.9 words for normal Japanese elderly aged 60–69 years and 7.2 words for those aged 70–79 (Ito et al., 2004). In the letter fluency test of the original ACE-R, 7 points were given for >17 words, 6 points for 14–17 words, 5 points for 11–13 words, 4 points for 8–10 words, 3 points for 6–7 words, 2 points for 4–5 words, and 1 point for 2–3 words. Therefore, we changed the scores of the Japanese phonological fluency test as follows: 7 points were given for >13 words, 6 points for 11–13 words, 5 points for 8– 10 words, 4 points for 6–7 words, 3 points for 4–5 words, 2 points for 3 words, and 1 point for 2 words.

**ITEM 5. FLUENCY (Animals)**

**
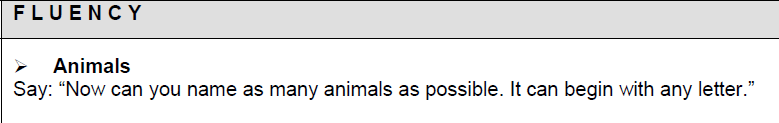
**

This item was translated directly and required no cultural adaptation.

**ITEM 6. MEMORY**

**
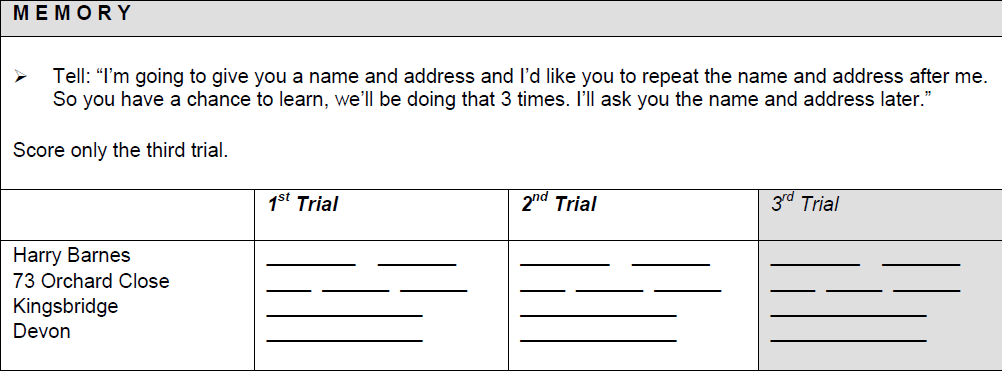
**

**Instructions for this item were translated directly.**

| Culturally Adapted Replacement | Language | Reasoning and Procedure |
| --- | --- | --- |
| Names most commonly found in the population were selected. The numbers of syllables and words for the names and address were also kept the same. | Malayalam^1^, Castillian Spanish^3^, Japanese^6^, French^13,14^, Peruvian Spanish^15^, Spanish^16^, Hindi, Hebrew, Greek^17^ | The length, familiarity and imaginability of the item were retained. |
| The name and address were replaced with a Chinese, Italian, Saudi Arabian Arabic, Korean, Cantonese Chinese, Slovak, Spanish, Danish, Saudi Arabian Arabic, Hungarian, Lithuanian name and address. | Chinese^4^, Italian^8^, Korean^9^, Saudi Arabian Arabic^11^, Cantonese Chinese^12^, Slovak^18^, Spanish^19, 20^, Danish^21^, Hindi, Polish, Hungarian, Lithuanian^22^ | The item had to be consistent with the countries’ systems. |
| Common German street names and less well-known towns were selected .eg. Frauenstrasse 24, Spremberg, Brandenburg. | German^23,24^ | The item’s original criteria were followed to prevent associations. |
| A common Egyptian name and street were selected with the street translating to ‘The Museum Road. | Egyptian Arabic | The name was common among both Christian and Muslim Egyptians.  The address had no peculiarities so it could be easier to remember. |
| The Welsh name ‘Harri Puw’ was chosen.  The address was changed to ‘Stryd y Berllan’ (Orchard Street), ‘Bontgoch’ (Little Red Bridge), ‘Gwynedd’. | Welsh | The original English name and address may not be familiar to older Welsh speakers.  The name ‘Harri’ is a Welsh equivalent for ‘Harry’. ‘Puw’ is a common Welsh surname which retained the number of syllables as the original.  There was no direct translation of ‘Close’. ‘Stryd y Berllan’ was a close translation.  The literal translation of ‘Kingsbridge’ would have twice as many syllables as the originals so ‘Bontgoch’ as it retained the number of syllables as the original.  ‘Gwynedd’ is a Welsh county which retained the number of syllables as the original. |
| The Hungarian name ‘Péter’ was chosen.  The street and the city for the address were changed to ‘Tavasz utca’ and ‘Gyöngyös’ respectively. | Hungarian | The original English names do not exist in Hungarian and majority of Hungarians do not speak English. The name ‘Péter’ is as common as ‘Harry’ in the UK.  For the address, ‘Tavasz utca’closely translates to ‘Market Street’ and the city of ‘Gyöngyös’ is around the same size as ‘Rockhampton’ and shares characteristics such as being a governmental area but not a large city. |
| Three versions of the item were created with a different address for each version. The terms ‘Shareaa’ (street), ‘Tareeq’ (road) and ‘Mamaer’ (passageway) were used across the three versions. | Saudi Arabian Arabic^11^ | This was consistent with the original assessment. |
| The address was given in the reverse order. | Persian^10^ | The address is given in reverse in Iran. |
| The scoring differed so that 1 point was given for prefecture, 1 point for city, 1 point for town, 2 points for block number and 2 points for name. | Japanese^7^ | The Japanese address had one more component to it than the original. |

**ITEM 7. MEMORY**

**
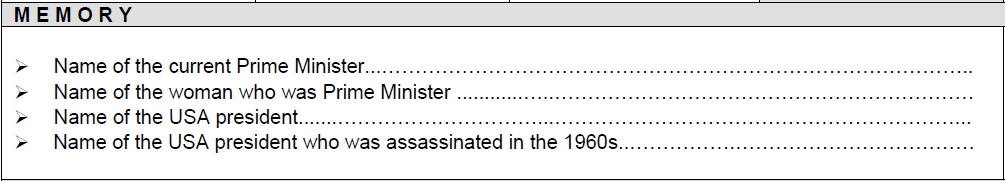
**

| Culturally Adapted Replacement | Language | Reasoning and Procedure |
| --- | --- | --- |
| ‘Name of the current Prime Minister’ was replaced:  ‘Name of the Indian capital’  ‘Name of the Prime Minister of Spain’  ‘Name of the Prime Minister of Japan’  ‘Name of the Prime Minister of Greece’  ‘Name of the President of the Italian Republic’  ‘Name of the president of Korea’  ‘Name of the current Prime Minister of Iran’  ‘Name of the current Chief Executive of Hong Kong SAR’  ‘Name of the president of Peru’  ‘Name of the current president of Slovakia’  ‘Name of the current chancellors’  ‘Name of the current president of Brazil’  ‘Name of the Prime Minister of Israel’ | Malayalam^1^, Castillian Spanish^3^, Japanese^5^, Greek^7^, Italian^8^, Korean^9^, Persian^10^, Cantonese Chinese^12^, Peruvian Spanish^15^, Slovak^18^, German^24^, Brazilian Portuguese^25^, Hebrew | The replacements chosen fit the cultural context of the target population.  ‘Prime Minister’ would be changed to ‘President’ to account for various government systems, such as in Korea.  The current political situation in Slovak is constantly changing and a presidential post is stable in comparison and so a more appropriate replacement.  Replacements for the Hebrew version reflected the political situation of Israel. |
| ‘Name of the woman who was Prime Minister’ was replaced:  ‘Name of the Indian currency’  ‘Name of Spain’s first democratically elected president’  ‘Name of the national singer of the Showa period’  ‘Name of the last Prime Minister’  ‘Name of the Prime Minister who nationalized the Iranian oil industry and the name of Iran’s Chief Justice who was assassinated by terrorists’  ‘Name the year of Hong Kong’s reversion to China’  ‘Name of the previous Pope of the Catholic Church’  ‘Name of the mayor of Lima’  ‘Name of the president of the Hellenic Republic’  ‘Name of the first president of Czechoslovakia after the Velvet revolution in 1989’  ‘Name of the previous chancellor’  ‘Name of the president who proposed and built the federal capital Brasilia in the early 50’s’  ‘Name of the president in the Republic of Hungary’  The answer was John Paul II for the Polish replacement.  ‘Name of the woman who was Prime minister of Israel’  The answer was Adolfo Suarez for the Spanish replacement. | Malayalam^1^, Castillian Spanish^3^, Japanese^5,7^, Italian^8^, Persian^10^, Cantonese Chinese^12^, Peruvian Spanish^15^, Spanish^16^, Greek^17^, Slovak^18^, German^24^, Brazilian Portuguese^25^, Hungarian, Polish, Hebrew | The replacements chosen fit the cultural context of the target population.  ‘The Pope of the Catholic Church’ is a far better known figure amongst the target population of Spain, as it is a Catholic country. This replacement also account for low educational profiles.  An important domestic event from the past had to be selected that was connected to revolutionary times in Slovakia and so is well known.  Hungary has both a Prime Minister and a president so this was an appropriate replacement.  The Polish replacement was selected to be equivalent to Margaret Thatcher.  Replacements for the Hebrew version reflected the political situation of Israel.  The Spanish replacement was selected to be equivalent to Margaret Thatcher and Adolfo Suarez is the first prime minister since the restoration of the democracy. |
| ‘Name of the USA president’ was replaced:  ‘Name of the State Chief’  ‘Name of the current president of the People’s Republic of China’  ‘Name of the former president of Peru’  ‘Name of the president of Israel’ | Malayalam^1^, Cantonese Chinese^12^, Spanish Peruvian^15^, Hebrew | The replacements chosen fit the cultural context of the target population.  Replacements for the Hebrew version reflected the political situation of Israel. |
| ‘Name of the USA president assassinated in the 1960’s’ was replaced:  ‘Name of the city where Taj Mahal is located’  ‘Name of the only female emperor in Chinese history’  ‘Name of the Japanese Prime Minister involved in the Lockheed bribery scandal’  ‘Name of the first president of the People’s Republic of China’  ‘Name of the minister of economy in Peru’  ‘Can you remember the date of the terrorist attack on the twin towers of the World Trade Centre in New York’  ‘Name of the US president’ | Malayalam^1^, Chinese^4^, Japanese^5^, Cantonese Chinese^12^, Peruvian Spanish^15^, Slovak^18^, Hebrew | The replacements chosen fit the cultural context of the target population.  The event of 11^th^ September was selected as it is universally known and would be recognized by target population of Slovak. This replacement is also culturally appropriate for future as it is will be historically applicable.  Replacements for the Hebrew version reflected the political situation of Israel. |
| ‘What is the capital city of the country where you live?’, ‘What is the name of the previous governor of your country?’, ‘Name of the president of the USA’, ‘Name of the current governor of your country’ | Saudi Arabian Arabic^11^ | The Saudi Arabian Arabic replacements chosen were designed to apply to different forms of government, culture and for different countries. It can be applies to kings, prime ministers, princes, sheikhs or presidents. In Saudi Arabia the king has the role of the prime minister and therefore it was the chosen replacement. |
| Appropriate Hindi questions replaced the original ones. | Hindi | The Hindi questions that replaced the original ones focused on current Indian political leaders. Questions took into account the political and social situations of India. |
| Appropriate Egyptian Arabic questions replaced the original ones. | Egyptian Arabic | The Egyptian Arabic questions that replaced the original ones focused on the Egyptian government system, where instead of the Prime Minister there is the President of Egypt. |

**ITEM 8. LANGUAGE**

**
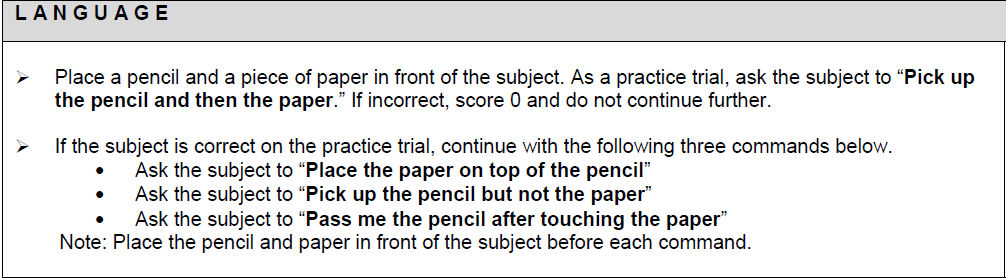
**

This item was translated directly and required no cultural adaptation.

**ITEM 9. LANGUAGE**

**
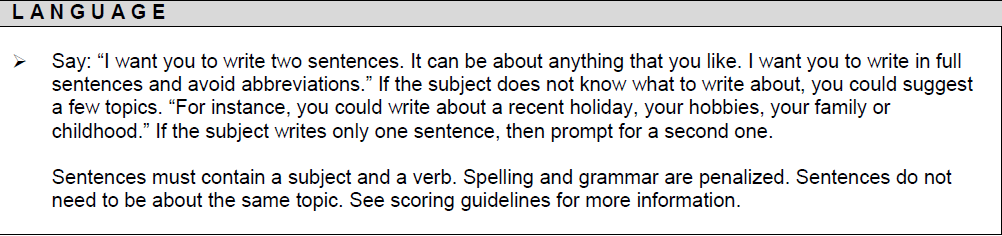
**

This item was translated directly and required no cultural adaptation.

**ITEM 10. LANGUAGE**

**
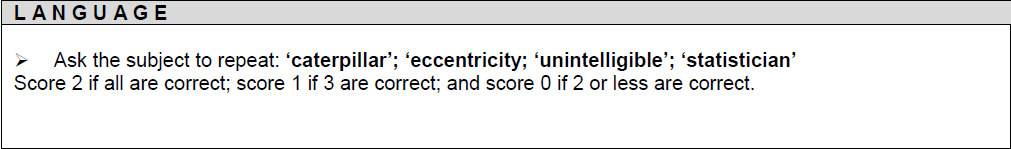
**

| Culturally Adapted Replacement | Language | Reasoning and Procedure |
| --- | --- | --- |
| Replacement words were chosen for Malayalam, Korean, French, Spanish, Greek, Slovak, German, Hebrew, Polish, Hungarian and Welsh.  The Egyptian Arabic words ‘Salsabil’ (any refreshing drink), ‘Khozabalat’ (something sarcastically mythical), ‘Shahbander’ (harbour master or wealthy merchant) and ‘Istishfaa’ (recovering) replaced the original words.  The Hindi words for ‘famous’, ‘encourage’, ‘ideal’ and ‘decade’ replaced the original words.  The Japanese words ‘kirigirisu’, ‘hototogisu’, ‘tororosoba’ and ‘ikijibiki’ replaced the original words.  The Chinese words for ‘weekend’, ‘contrast’, ‘rock and roll band’ and ‘hiding one’s head in the sand’ replaced the original words. | Malayalam^1^, Korean^9^, French^13,14^, Spanish^16^, Greek^17^, Slovak^18^, German^24^, Hebrew, Polish, Hungarian, Welsh, Egyptian Arabic, Hindi, Japanese^5,6,7^, Cantonese Chinese^12^. | Words chosen to replace the original words retained the length, frequency and difficulty to articulate. |
| Chinese sayings replaced the original words. | Chinese^4^ | The sayings chosen fit the cultural context of the target population. |
| ‘Caterpillar’ was replaced with ‘Cucaracha’. | Castillian Spanish^3^ |  |
| The word ‘eccentricity’ was replaced with the Welsh word ‘unigolyddol’ (individualistic). | Welsh | The Welsh replacement retained the number of syllables. |
| The Saudi Arabian Arabic words for ‘will deal with them on your behalf’, ‘forcing them to comply’, ‘giving water of life’ and ‘passing on through generations’ replaced the original words. | Saudi Arabian Arabic^11^ | Saudi Arabian Arabic consists primarily of words with just one or two syllables. The Holy Quran had to be searched to find four words with three syllables to retain the same number as the original words. |

**ITEM 11. LANGUAGE**

**
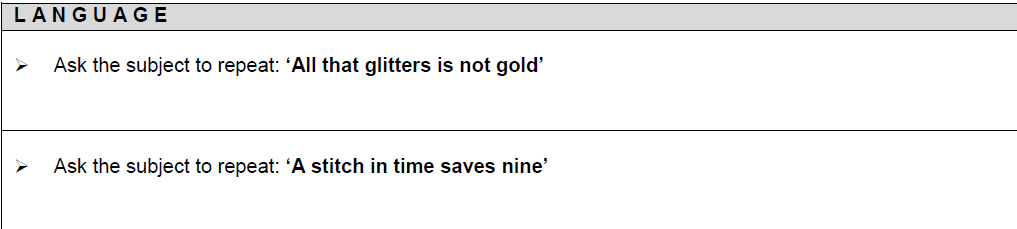
**

| Culturally Adapted Replacement | Language | Reasoning and Procedure |
| --- | --- | --- |
| Replacement sayings were chosen for Castillian Spanish, French, Greek, Spanish and Hungarian.  The Egyptian Arabic sayings ‘only stretch your legs as much as your duvet allows’ and ‘one hand can’t clap’ replaced the original sayings.  The Hindi saying ‘What does a monkey know about the taste of ginger?’ replaced one of the original sayings. | Castillian Spanish^3^, French^13,14^, Greek^17^ Spanish^19^, Hungarian, Egyptian Arabic, Hindi | Sayings chosen to replace the original ones retained the length, frequency and difficulty to articulate. |
| Chinese sayings replaced the original sayings.  Korean sayings replaced the original sayings.  Persian sayings replaced the original sayings.  Cantonese Chinese sayings replaced the original sayings.  ‘The purple porridge has peaches and plums’ is a Spanish Peruvian saying that replaced an original saying. | Chinese^4^, Korean^9^, Persian^10^, Cantonese Chinese^12^, Peruvian Spanish^15^ | The sayings chosen fit the cultural context of the target population.  The Cantonese Chinese saying that replaced the original was common in Taiwan and the People’s Republic of China. |
| ‘The orchestra played and audience applauded’ is a Hebrew saying that replaced the original saying. | Hebrew | The original saying, when translated into Hebrew made no sense so a culturally appropriate saying had to be chosen as a replacement. |
| A Polish saying was chosen to replace ‘a stitch in time saves nine’. | Polish | The Polish saying that was chosen retained a rhyme similar to the ‘time’ and ‘nine’ rhyme. |
| A Saudi Arabian Arabic sentence that retains the meaning of a ‘stitch in time saves nine’ was chosen to replace it. | Saudi Arabian Arabic^11^ | The Saudi Arabian Arabic saying retained the meaning of the original saying. |
| ‘I’r pant y rhed y dŵr’ (to the hollow runs the watter: the rich get richer) replaced ‘a stitch in time saves nine’. | Welsh | The Welsh proverb that closely relates to ‘a stitch in time saves nine’ has more syllables than the original saying and would be more challenging in terms of listening, processing and articulation. Therefore, a more appropriate saying was chosen to replace the original. |

**ITEM 12. LANGUAGE**


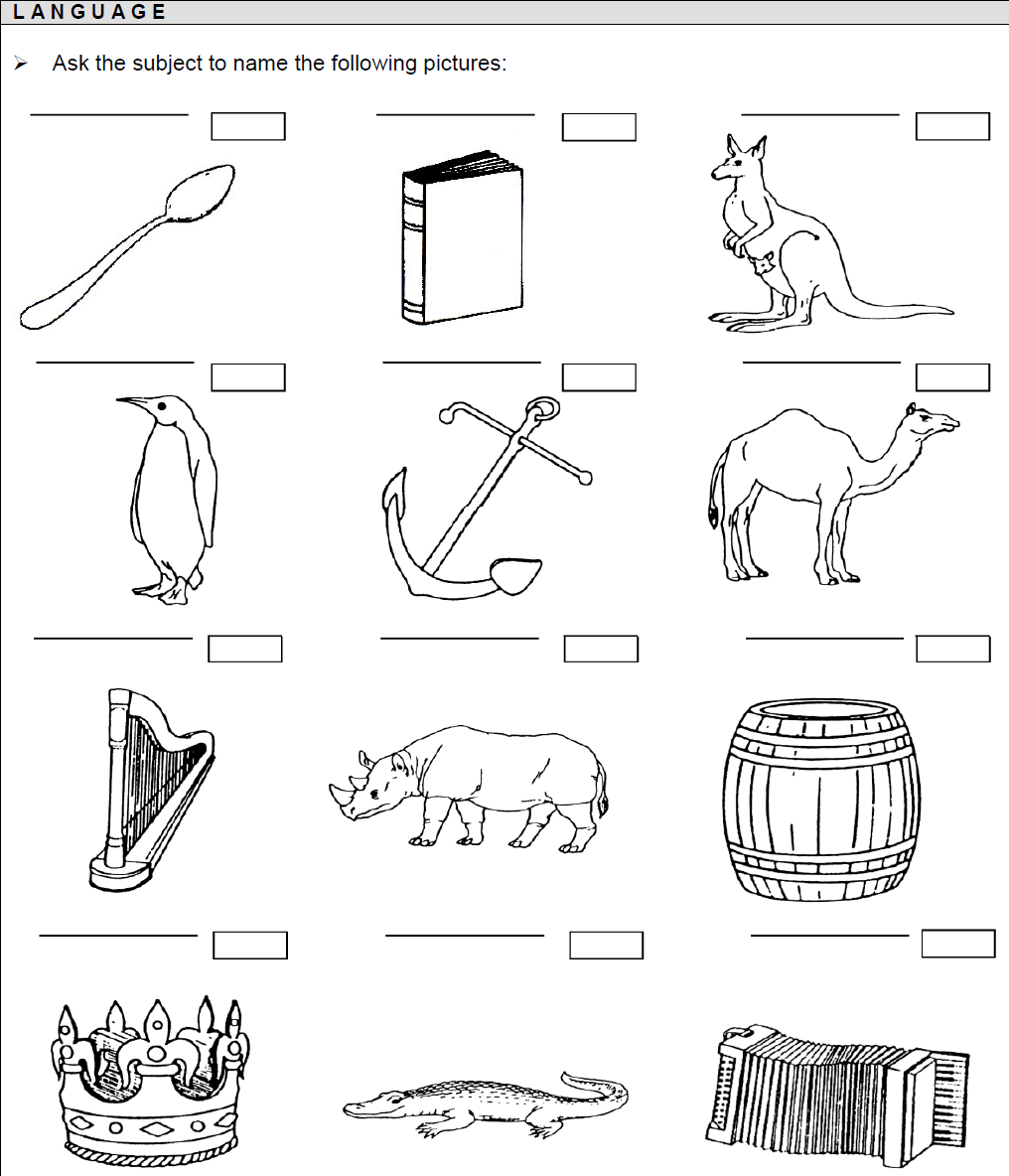


| Culturally Adapted Replacement | Language | Reasoning and Procedure |
| --- | --- | --- |
| The ‘kangaroo’ was replaced:  With a ‘zebra’.  With a ‘sea horse’.  With a ‘giraffe’.  With a ‘squirrel’.  With a ‘goat’. | Malayalam^1^, Japanese^5^, Persian^10^, Egyptian Arabic, Polish, Hindi | The original pictures were less familiar to the Japanese.  A ‘kangaroo’ is not widely known amongst Egyptian individuals so a picture of a ‘giraffe’ was selected.  A ‘kangaroo’ is considered an Australian animal. A Polish animal would be a ‘squirrel’.  A ‘kangaroo’ is not found or seen in India, but a ‘goat’ is quite common. |
| The ‘penguin’ was replaced:  With an ‘owl’.  With a ‘parrot’.  With a ‘candle’. | Japanese^5^, Persian^10^, Egyptian Arabic, Hindi | The original pictures were less familiar to the Japanese.  A ‘penguin’ is not widely known amongst Egyptian individuals so a picture of a ‘parrot’ was selected.  A ‘penguin’ is not found or seen in India, but a ‘candle’ is a common object found there which fits the cultural context. |
| The ‘anchor’ was replaced:  With a ‘light bulb’.  With a ‘flag’. | Egyptian Arabic, Hindi | An ‘anchor’ is not widely known amongst Egyptian individuals so a picture of a ‘light bulb’ was selected.  An ‘anchor’ is not found or seen in India, but a ‘flag’ is a common object. |
| The ‘harp’ was replaced:  With a ‘cigar’.  With a ‘trumpet’.  With a ‘santoor’.  With a ‘violin’.  With a ‘violin’. | Japanese^5,7^, Persian^10^, Greek^17^, Egyptian Arabic, Hindi | The original pictures were less familiar to the Japanese.  The ‘trumpet’ was selected for the Japanese version according to the original criteria.  A picture of a ‘santoor’, a stringed musical instrument, would be more familiar to people in the targeted geographical region.  Elderly individuals in Greece may not be familiar with a harp but would recognize a violin.  A ‘harp’ is not widely known amongst Egyptian individuals so a picture of a ‘violin’ was selected.  A ‘harp’ is not a familiar musical instrument in India but a sickle is a common Indian tool. |
| The ‘rhino’ was replaced:  With a ‘lion’.  With a ‘giraffe’. | Egyptian Arabic, Hindi | A ‘rhino’ is not widely known amongst Egyptian individuals so a picture of a ‘lion’ was selected.  A ‘giraffe’ is a common animal because of its visibility to people in Indian zoos. |
| The ‘barrel’ was replaced:  With a ‘candle’.  With a ‘baby carriage’.  With a ‘light bulb’.  With a ‘box’.  With an ‘Indian drum’. | Malayalam^1^, Japanese^5,7^, Persian^10^, Hindi | The original pictures were less familiar to the Japanese.  The ‘light bulb’ was selected according to the original criteria.  A ‘barrel’ is not common in India, but an ‘Indian drum’ is a common musical instrument. |
| The ‘crown’ was replaced:  With an ‘umbrella’. | Hindi | A ‘crown’ is not commonly recognised in India, but an ‘umbrella’ is universally used. |
| The ‘crocodile’ was replaced:  With a ‘peak’. | Japanese^5^ | The original pictures were less familiar to the Japanese. |
| The ‘accordion’ was replaced:  With a ‘setar’.  With a ‘pig’. | Persian^10^, Hindi | A picture of a ‘setar’, a stringed musical instrument, would be more familiar to people in the targeted geographical region.  An ‘accordian’ is not an appropriate object in Indian context. |
| The ‘alligator’ was accepted as a ‘crocodile’ or lizard’ and the harp was accepted as a ‘lyre’. | Czech^2^ | A picture may have alternative names. |
| Apart from the watch, pencil and camel, the remaining nine items were all changed. | Saudi Arabian Arabic^11^ | The original pictures had to be changed as it they were found to be too unfamiliar to the Saudi Arabian society in early testing. They were replaced with pictures of items that were neither highly familiar or very infrequent. |
| Pictures were changed account for cultural influences. | Hindi | Pictures that were used to replace the original ones accounted for Indian language, familiarity, image agreement and visual complexity. |

**ITEM 13. LANGUAGE**

**
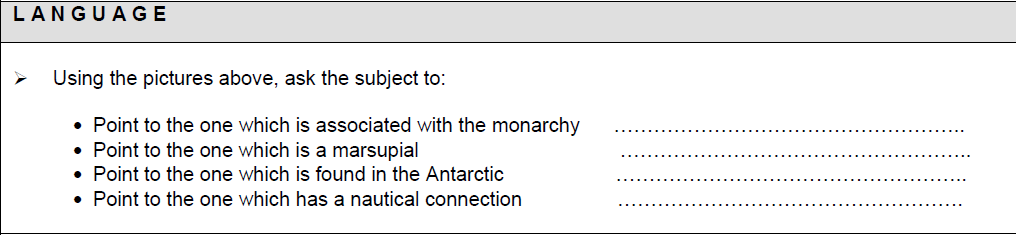
**

| Culturally Adapted Replacement | Language | Reasoning and Procedure |
| --- | --- | --- |
| Changes to questions were made in the Japanese, Persian | Japanese^5^, Persian^10^ | Changes were made according to changes in images in Item 12: Language |
| ‘Point to the one which is associated with the monarchy’ was replaced:  ‘Which thing is used for filling with water?’ | Chinese^4^ | The question was changed in the Chinese version according to the change in the corresponding image in Item 12: language. |
| ‘Point to the marsupial’ was replaced:  ‘Point to the reptile’  ‘Which animal lives in Australia?’  ‘Point to the thing that flies’  ‘Point to the rodent’ | Castillian Spanish^3^, Chinese^4^, Egyptian Arabic, Polish | The Spanish elderly population may not be familiar with the term ‘marsupial’ as it is common in Australia but not in Spain.  The question was changed in the Egyptian Arabic version according to the change in the corresponding image in Item 12: Language.  The question was changed in the Polish version according to the change in the corresponding image in Item 12: Language. |
| ‘Point to the one which is found in the Antarctic’ was replaced:  ‘Point to the thing which is present in the desert’ | Egyptian Arabic | The question was changed in the Egyptian Arabic version according to the change in the corresponding image in Item 12: Language. |
| ‘Point to the one which has a nautical connection was replaced:  ‘Point to the thing that is found in the river Nile’ | Egyptian Arabic | The question was changed in the Egyptian Arabic version according to the change in the corresponding image in Item 12: Language. |
| ‘Point to the figure found at Pantanal’ was chosen to replace one of the tasks. | Brazilian Portuguese^25^ | ‘Pantanal’ is a swamp area that is a well known Brazilian ecosystem and is therefore culturally appropriate for the target population. |

**ITEM 14. LANGUAGE**

**
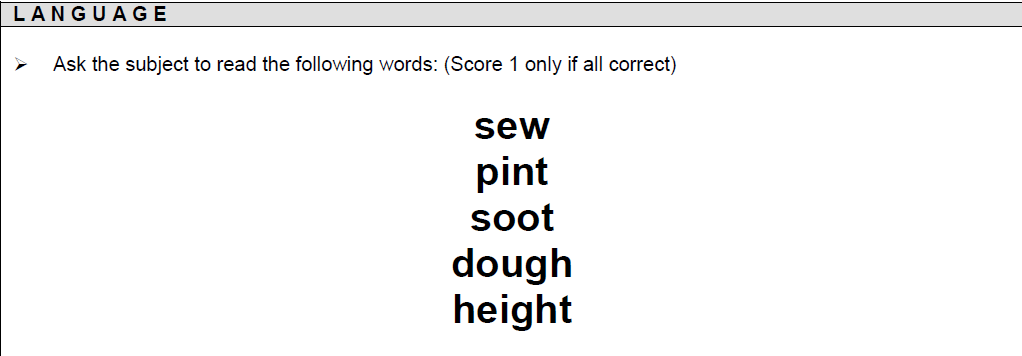
**

| Culturally Adapted Replacement | Language | Reasoning and Procedure |
| --- | --- | --- |
| Appropriate replacements were selected for the irregular words in Malayalam, Japanese, Korean, Persian, French, Greek, Spanish and Hungarian | Malayalam^1^, Japanese^6^, Korean^9^, Persian^10^, French^13,14^, Greek^17^, Spanish^19^, Hungarian | Length and frequency of the irregular words were taken into account when selecting words from the language. |
| The irregular words were replaced with the Brazilian Portuguese words ‘taxi’ (taxi), ‘testa’ (forehead), ‘saxofone’ (saxophone), ‘fixar’ (to fix) and ‘ballet’ (ballet). | Brazilian Portuguese^25^ | The Brazilian Portuguese words selected were of similar difficulty to the original words. |
| Japanese kanji compounds (ideographic script) replaced the original irregular words. | Japanese^5,7^ | Japanese words are expressed in kanji compounds that are equivalent to irregular words. |
| Exceptional Cantonese Chinese characters replaced the original irregular words. | Cantonese Chinese^12^ | The Cantonese Chinese characters that were selected did no obey the normal sound-to-print rules of the language. |
| Spanish words selected from the Word Accentuation Test (WAT) replaced the original irregular words. | Spanish^16^ | Irregular words are not common in Spanish. |
| The original irregular words were replaced with the Welsh words ‘cwyn’, ‘ynghyd’, ‘englyn’, ‘hwyr’ and ‘pryfn’. | Welsh | Irregular words are not common in Welsh. The two main issues in pronouncing Welsh words were identified: words with dipthongs and words where the vowel needs to be lengthened without a cue given. ‘Cwyn’ and ‘hwyr’ has two versions, where the ‘w’ or ‘y’ is stressed. ‘Ynghyd’ requires pronouncing the ‘y’ long. ‘Englyn’ requires pronouncing the ‘y’ short. ‘Pryfyn’ has two pronunciations. |
| Polish words that are difficult to read due to various other reasons replaced the original irregular words. | Polish | The original irregular words are too obvious in Polish. The words chosen to replace them were difficult to read for other reasons, such as containing diagraphs, where the letters are read together or separately depending on the word (‘cz’ or ‘rz’) or words in which the beginning can be omitted .eg. re/produkcja. |
| The irregular words were replaced with ‘Hollywood’, ‘vedette’, ‘blues’, ‘tour’ and ‘acapella’.  Italian words with irregular accentuation replaced the original irregular words.  Hebrew words that come from less frequent words forms replaced the original irregular words.  Slovak words with irregular accentuation, mutation or grammatical exceptions replaced the original irregular words.  The irregular words were replaced with ‘aggregation’, ‘beginning’, ‘distress’, ‘sage’ and ‘desire’. | Castillian Spanish^3^, Italian^8^, Hebrew, Slovak^18^, Hindi, Spanish | There are no irregular words in Castillian Spanish so foreign words that would be easily recognized by the target population were selected as replacements.  There are no irregular words in Italian so words with irregular accentuation were selected as replacements.  There are no irregular words in Hebrew so less frequent word forms were selected as replacements.  There are no irregular words in Slovak so words with irregular accentuation, mutation or grammatical errors that are commonly spoken and written and are well known by the target population were selected.  There are no irregular words in Hindi and the literal translation from English to Hindi resulted in inappropriate words for the task. Regular Hindi words of comparable frequency and word length to the original words were selected. |
| The irregular words were replaced with the Arabic words ‘feema’ (what or whole), ‘amma’ (about what), ‘yaseen’ (the name of a prophet from the Holy Quran), ‘tawoos’ (the peacock) and ‘elah’ (God). | Saudi Arabian Arabic^11^ | The Saudi Arabian Arabic words chosen do not obey the normal sound-to-print rules and correct pronunciation is indicated by diacritics (symbols written above the word). |
| The irregular words were replaced with the Arabic words ‘Yes’ (a name), ‘hatti’ (until), ‘amro’ (a name), ‘kathalik’ (and so on) and ‘namoa’ (they have slept). | Egyptian Arabic | Translating the original irregular words into Egyptian Arabic would not result in irregular words so other words had to be identified as replacements. |

**ITEM 15. VISUOSPATIAL ABILITIES (Infinity Diagram)**

**
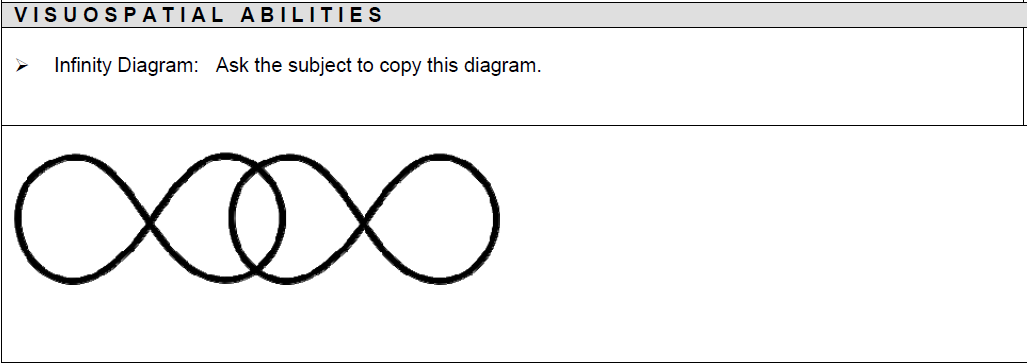
**

This item was translated directly and required no cultural adaptation.

Note: In the Saudi-Arabian Arabic version the location of the figure was placed on the right hand side as Arabic is read from right to left^11^

**ITEM 15. VISUOSPATIAL ABILITIES (Wire Cube)**

**
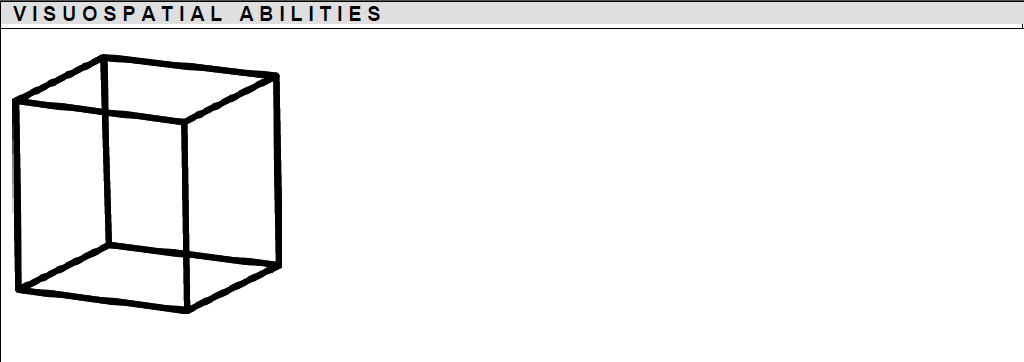
**

This item was translated directly and required no cultural adaptation.

Note: In the Saudi-Arabian Arabic version the location of the figure was placed on the right hand side as Arabic is read from right to left^11^

**ITEM 15. VISUOSPATIAL ABILITIES (Clock)**

**
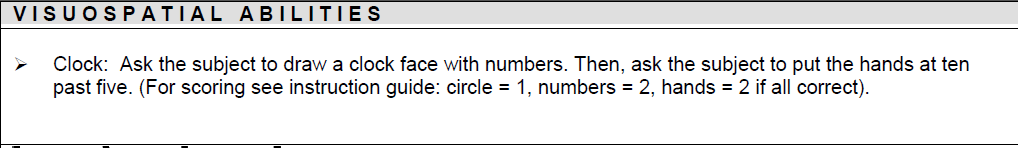
**

Instructions for this item were translated directly.

| Culturally Adapted Replacement | Language | Reasoning and Procedure |
| --- | --- | --- |
| The subject was asked to put the hands at ten past eleven. | Italian^8^, Peruvian Spanish^15^ | This specific time is reported to be the most sensitive for detecting cognitive impairment. |

**ITEM 16. VISUOSPATIAL ABILITIES**

**
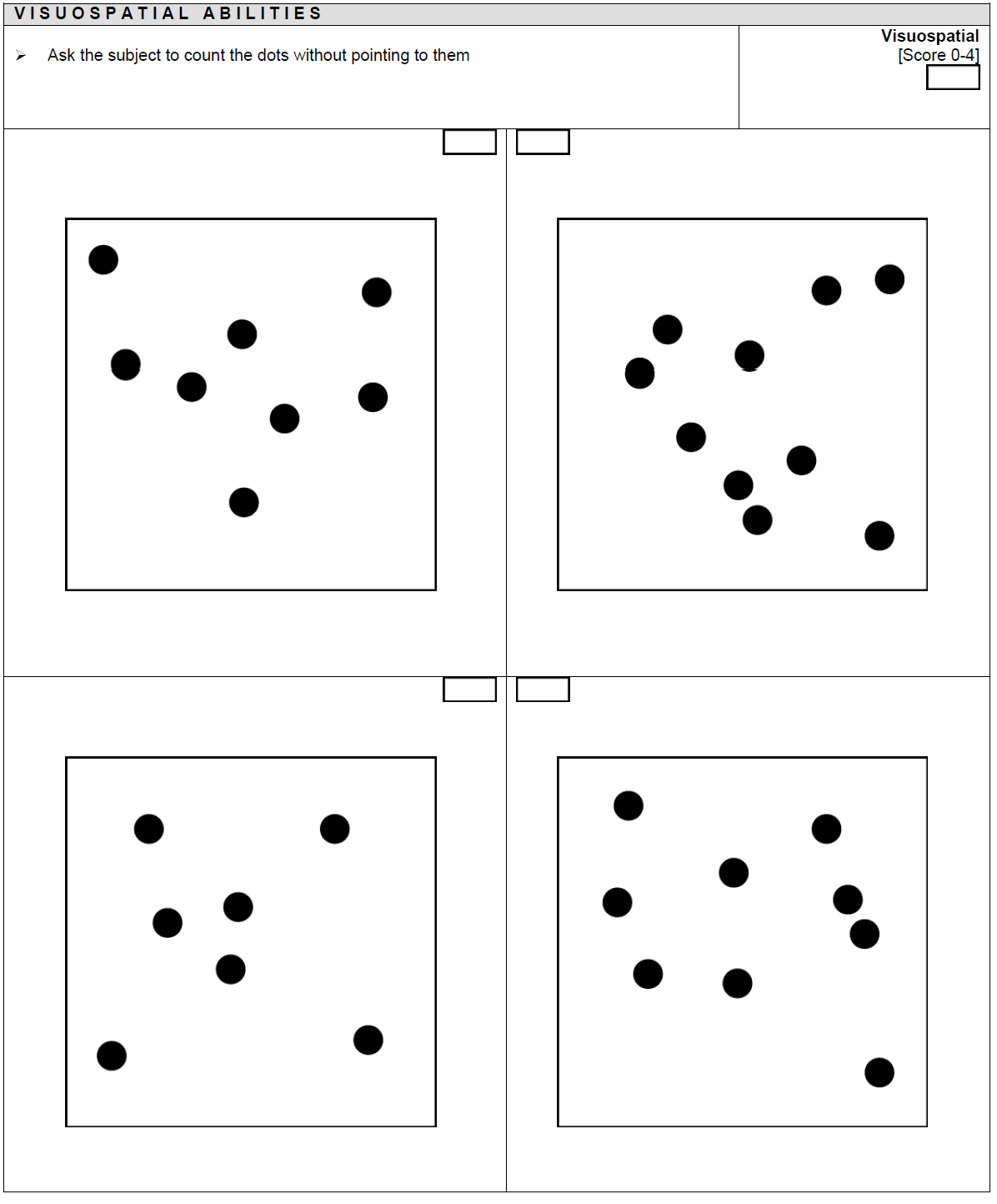
**

This item was translated directly and required no cultural adaptation.

Note: In the Saudi-Arabian Arabic version the location of the figure was placed on the right hand side as Arabic is read from right to left^11^

**ITEM 17. VISUOSPATIAL ABILITIES**

**
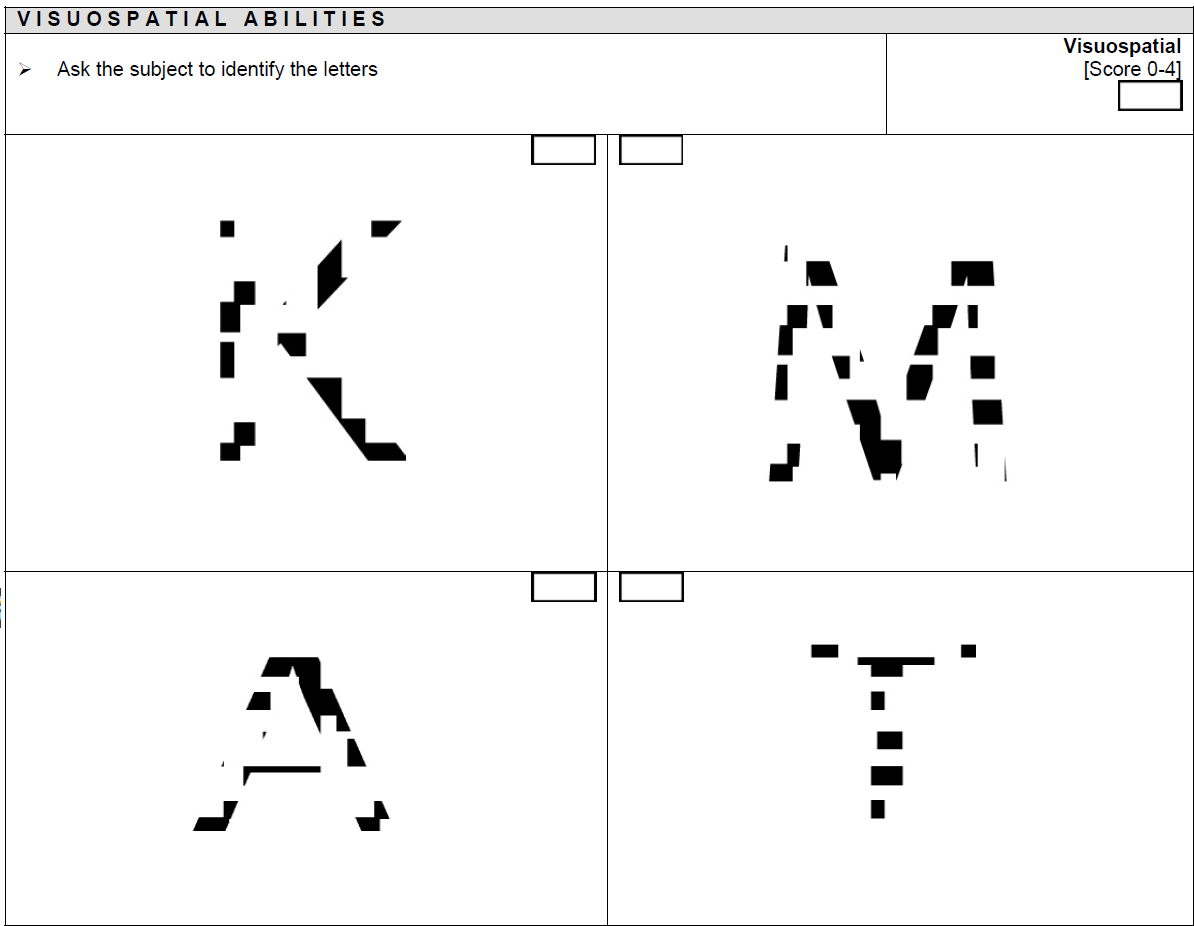
**

Instructions for this item were translated directly.

| Culturally Adapted Replacement | Language | Reasoning and Procedure |
| --- | --- | --- |
| Four Katakana letters (Japanese syllabograms) replaced the English letters. | Japanese^5,7^ | Katakana letters were used as the Japanese elderly may not be familiar with English letters. The chosen Katakana letters had similar geometric complexity as the original letters. |
| Four Chinese characters (Chinese radicals) replaced the English letters. | Cantonese Chinese^12^ | The chosen Chinese characters were 70% fragmented according to the Visual Object and Space Perception Battery (VOSP). |
| ‘K’ was replaced with ‘R’. | Italian^8^ | There is no letter ‘K’ in the Italian alphabet. |
| The Saudi Arabian Arabic equivalent of the letters ‘M’, ‘L’, ‘H’ and ‘W’ were used. | Saudi Arabian Arabic^11^ | The Arabic letters that were selected had to be completely distinguishable from other letters and to have no dots. Any fragmentation of the letters could not create the illusion that there are dots over or under the letters. |
| Egyptian Arabic letters were used. | Egyptian Arabic | The complexity of the letter’s shape and degree of obscurity mattered more than the sound.  The Arabic letters had to be distinguishable from each other and have no dots as they could be confused with un-dotted letters. This could be due to poor eye sight. |
| The Hindi equivalents of the original letters were used. | Hindi | The Hindi equivalents of the original letters had similar phonetic sounds and were found appropriate in pilot testing. |

**ITEM 18. MEMORY**

**
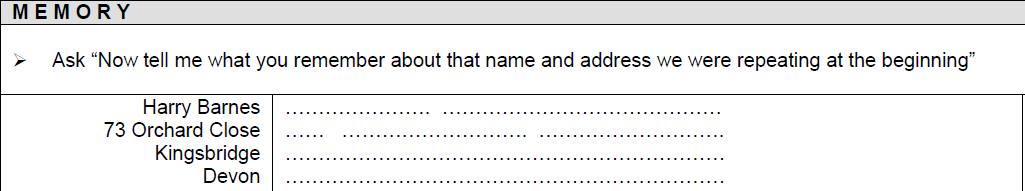
**

Refer to Item 6: Memory.

**ITEM 19. MEMORY**

**
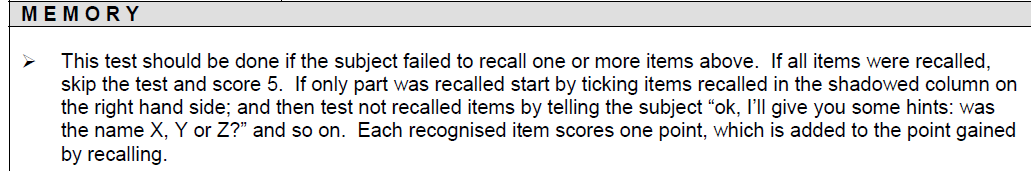
**

Refer to Item 6: Memory.

| Culturally Adapted Replacement | Language | Reasoning and Procedure |
| --- | --- | --- |
| Alternative names and addresses were in Portuguese, Italian Greek, Spanish, Hindi | Portuguese^3^, Italian^8^, Greek^17^, Spanish, Hindi | Alternative names and addresses were phonetically and semantically similar to the originals, with similar syllable length. |
| The alternative address numbers were 24 and 46.  Alternative Welsh cities and county names were used. | Hungarian | The number 24 is the reverse of 42 and the number 46 has the same same number in the tens place.  Similar sized cities to ‘Gyönygös’ were chosen, one nearby and one on the opposite part of the country. |

**REFERENCES**

**1.** Mathuranath PS, Hodges JR, Mathew R, Cherian PJ, George A, Bak TH. Adaptation of the ACE for a Malayalam speaking population in southern India. Int J Geriatr Psychiatry. 2004;19:1188-94.

**2.** Bartoš A, Raisová M, Kopeček M. Novelizace české verze Addenbrookského kognitivního testu (ACE-CZ). *Česká a Slovenská Neurologie a Neurochirurgie*. 2011;74:681-4.

**3.** Matias-Guiu, J.A., Fernandez de Bobadilla, R., Escudero, G., Perez-Perez, J., Cortes, A., Morenas-Rodriguez, E., Valles-Salgado, M., Moreno-Ramos, T., Kulisevsky, J., & Matias-Guiu, J. (2015). Validation of the Spanish version of Addenbrooke’s cognitive examination III for diagnosing dementia. Neurologia, 30, pp. 545-551.

**4.** Fang R, Wang G, Huang Y et al. Validation of the Chinese version of Addenbrooke’s cognitive examination-revised for screening mild Alzheimer’s disease and mild cognitive impairment. Dement Geriatr Cogn Disord. 2014;37:223-31 [Erratum Dement Geriatr Cogn Disord. 2015;39:91].

**5.** Dos Santos Kawata KH, Hashimoto R, Nishio Y et al. A validation study of the Japanese version of the Addenbrooke’s Cognitive Examination-Revised. Dement Geriatr Cogn Dis Extra. 2012;2:29-37.

**6.** Yoshida H, Terada S, Honda H et al. Validation of Addenbrooke’s cognitive examination for detecting early dementia in a Japanese population. Psychiatry Res. 2011;185:211-4.

**7.** Yoshida H, Terada S, Honda H et al. Validation of the revised Addenbrooke’s Cognitive Examination (ACE-R) for detecting mild cognitive impairment and dementia in a Japanese population. Int Psychogeriatr. 2012;24:28-37.

**8.** Pigliautile M, Ricci M, Mioshi E et al. Validation study of the Italian Addenbrooke’s Cognitive Examination Revised in a young-old and old-old population. Dement Geriatr Cogn Disord. 2011;32:301-7.

**9.** Heo JH, Lee KM, Park TH, Ahn JY, Kim MK. Validation of the Korean Addenbrooke’s Cognitive Examination for diagnosing Alzheimer’s dementia and mild cognitive impairment in the Korean elderly. Appl Neuropsychol Adult. 2012;19:127-31.

**10.** Pouretemad HR, Khatibi A, Ganjavi A, Shams J, Zarei M. Validation of Addenbrooke’s cognitive examination (ACE) in a Persian-speaking population. Dement Geriatr Cogn Disord. 2009;28:343-7.

**11.** Al Salman A.S. (2013) The Saudi Arabian Adaptation of the Addenbrooke’s Cognitive Examination – Revised (Arabic ACE-R). University of Glasgow Thesis.

**12.** Wong L, Chan C, Leung J et al. A validation study of the Chinese-Cantonese Addenbrooke’s Cognitive Examination Revised (C-ACER). Neuropsychiatr Dis Treat. 2013;9:731-7.

**13.** Bier JC, Ventura M, Donckels V et al. Is the Addenbrooke’s Cognitive Examination effective to detect frontotemporal dementia? J Neurol. 2004;251:428-31.

**14.** Bastide L, De Breucker S, van den Berge M, Fery P, Pepersack T, Bier JC. The Addenbrooke’s Cognitive Examination Revised is as effective as the original to detect dementia in a French-speaking population. Dement Geriatr Cogn Disord. 2012;34:337-43.

**15.** Custodio N, Lira D, Montesinos R, Gleichgerrcht E, Manes F. Usefulness of the Addenbrooke’s Cognitive Examination (Spanish version) in Peruvian patients with Alzheimer’s disease and Frontotemporal Dementia [in Spanish]. Vertex. 2012;23:165-

**16.** Garcia-Caballero A, Garcia-Lado I, Gonzalez-Hermida J et al. Validation of the Spanish version of the Addenbrooke’s Cognitive Examination in a rural community in Spain. Int J Geriatr Psychiatry. 2006;21:239-45.

**17.** [Konstantinopoulou E](http://www.ncbi.nlm.nih.gov/pubmed?term="Konstantinopoulou E"%5BAuthor%5D), [Kosmidis MH](http://www.ncbi.nlm.nih.gov/pubmed?term="Kosmidis MH"%5BAuthor%5D), [Ioannidis P](http://www.ncbi.nlm.nih.gov/pubmed?term="Ioannidis P"%5BAuthor%5D), [Kiosseoglou G](http://www.ncbi.nlm.nih.gov/pubmed?term="Kiosseoglou G"%5BAuthor%5D), [Karacostas D](http://www.ncbi.nlm.nih.gov/pubmed?term="Karacostas D"%5BAuthor%5D), [Taskos N](http://www.ncbi.nlm.nih.gov/pubmed?term="Taskos N"%5BAuthor%5D). Adaptation of Addenbrooke’s Cognitive Examination-Revised for the Greek population. Eur J Neurol. 2011;18:442-7.

**18.** Gondova (2012). Translation and adaptation of the Addenbrooke’s Cognitive Examination – Revised (ACE-R) in the diagnosis of subcortical dementia and Alzheimer disease. Neuropsychologi Dev Cogn B Aging Neuropsychol Cogn. 2015; 22:473-85.

**19.** Sarasola D, De Lujan M, Sabe L, Caballero A, Manes F. Utilidad del Addenbrooke’s Cognitive Examination en Espanol para el diagnostico de demencia y para la differenciacion entre la enfermidad de Alzheimer y la demencia frontotemporal [in Spanish]. Arg Neuropsicol. 2004;4:1-11.

**20.** Torralva T, Roca M, Gleichgerrcht E, Bonifacio A, Raimondi C, Manes F. Validation of the Spanish version of the Addenbrooke’s Cognitive Examination-Revised (ACE-R). Neurologia. 2011;26:351-6.

**21.** Stokholm J, Vogel A, Johannsen P, Waldemar G. Validation of the Danish Addenbrooke’s Cognitive Examination as a screening test in a memory clinic. Dement Geriatr Cogn Disord. 2009;27:361-5.

**22.** Margevičiūtė R, Bagdonas A, Butkus K et al. Adenbruko kognityvinio tyrimo metodikos – taisytos adaptacija lietuviškai kalbantiems gyventojams (ACE-RLT). Neurologijos seminarai. 2013;1(55):29-51.

**23.** Alexopoulos P, Greim B, Nadler K, Martens U, Krecklow B, Domes G, Herpertz S, Kurz A. Validation of the Addenbrooke’s Cognitive Examination for detecting early Alzheimer’s disease and mild vascular dementia in a German population. Dement Geriatr Cogn Disord. 2006;22:385-91.

**24.** Alexopoulos P, Ebert A, Richter-Schmidinger T et al. Validation of the German revised Addenbrooke’s cognitive examination for detecting mild cognitive impairment, mild dementia in Alzheimer’s disease and frontotemporal lobar degeneration. Dement Geriatr Cogn Disord. 2010;29:448-56.

**25.** Carvalho VA, Barbosa MT, & Caramelli, P. (2007). Brazilian adaptation of the Addenbrooke’s Cognitive Examination Revised. Neuropsychologia, 2, 212-216.
