# Supplementary material for: Developing and implementing guidelines on culturally adapting the Addenbrooke’s cognitive examination version III (ACE-III): a qualitative illustration
Source: BMC Psychiatry. 2020 Oct 6;20:492. doi: 10.1186/s12888-020-02893-6 (PMC7539399; doi:10.1186/s12888-020-02893-6)
Supplement: Supplementary file 3 — Additional file 3: Supplementary Material- Appendix A3. Table on ‘Proposed ACE-III questions developed from the guidelines’. [file 12888_2020_2893_MOESM3_ESM.doc]

Appendix A2

*Proposed ACE-III questions developed from the guidelines*

| **Proposed suggestions** | **Justification** | | |
| --- | --- | --- | --- |
| **1:Attention** |  | | |
| 1. The Urdu word for ‘season’ also means ‘weather’ so ask the question ‘which of the four seasons is it?’ 2. The Urdu words for ‘hospital’ and ‘county’ being used are too complex so ‘Hospital’ and ‘county’ will be spelt using Urdu letters. | 1. We are specifically referring to seasons, as the Urdu word for ‘season’ cannot be mistaken for ‘weather’ in the context of this question. 2. The use of the English words ‘hospital’ and ‘county’, spelt using Urdu letters, is common among British Urdu speakers.   Dates will only be accepted according to the English calendar as this is what is followed by majority of British Urdu speakers and is employed in their everyday use. | | |
| **2: Attention** |  | | |
| 1. ‘Key’ has one syllable while its Urdu translation has tw so ‘Key’ can be replaced with ‘bell’, spelt using Urdu letters. 2. ‘Lemon’ is directly translated into Urdu. 3. ‘Ball’ is spelt out using Urdu letters. | 1. The Urdu translation of ‘key’ has two syllables so we considered ‘bell’ as it is also related to the concept of a ‘door’. The Urdu translation of ‘bell’ also has two syllables but ‘bell’, spelt with Urdu letters, is commonly used among British Urdu speakers. 2. The Urdu translation for ‘lemon’ has one syllable and is therefore an ideal version. 3. The Urdu word for ‘ball’ is also one syllable but it was decided that the word ‘ball’, spelt using Urdu letters, is used more commonly by British Urdu speakers. | | |
| **3: Attention** |  | | |
| 1. Use the word ‘minus’ spelt using Urdu letters or the Urdu translation for ‘take away’. | 1. The term ‘minus’, spelt using Urdu letters, is commonly used as is the Urdu translation of the mathematical phrasing ‘take-away’, which is used to indicate subtraction. | | |
| **4: Memory: Refer to Question 2: Attention** |  | | |
| **5a: Fluency (Letters)** |  | | |
| 1. Replace the letter ‘P’ with the Urdu letter چ (chay) or گ (gaaf). | 1. It was initially proposed that we also use the phonetic equivalent of the letter ‘P’, پ (pay) but once the guideline’s rationale was used there was a debate between چ (chay) or گ (gaaf) as they both have the same frequency as the letter P across the Urdu language. | | |
| **6: Memory** |  | | |
| 1. The first name Afzal, Haroon, Kashif or Rasheed is used. The last name Butt or Khan is used. 2. The original ACE-III address will be retained and spelt using Urdu letters. | 1. These names were proposed as they are commonly known names amongst the British Urdu speaking community. 2. As the original ACE-III address is based in the UK it was considered appropriate for use as it was, spelt out using Urdu letters. | | |
| **7: Memory** |  | | |
| 1. The first question and third question of the original ACE-III, ‘Name of the current Prime Minister’ and ‘Name of the USA president’ are retained.   The second and fourth questions will be replaced with ‘Name of the British currency’, ‘Name of the city where (a ‘Wonder of the World’) is located’. | 1. The first question refers to the present government of the UK and it was decided that this would be known by all British Urdu speakers.   The second question was considered too difficult for British Urdu speakers. They would be more likely to know about the political history of their country of ethnicity as opposed to the UK. ‘Name of the British currency’ was proposed using the guidelines.  The third question was considered suitable for the British Urdu speaking population. The USA’s political situation was considered to have global prominence and therefore their president’s name would be common knowledge.  The fourth question was considered too difficult for British Urdu speakers as they would be more likely to know about the political history of their country of ethnicity. As this question did not relate to this or the government of the UK other suggestions were proposed. ‘Name of the city where Big Ben is located’ was deemed too easy a question, especially in comparison to the original. Naming the city of another ‘Wonder of the World’ such as the ‘Leaning Tower of Pisa’ was suggested as it is slightly more difficult. | | |
| **10: Language** |  | | |
| 1. ‘Eccentricity’ is replaced with   بازی نشانہ or  وزیر اعظم   1. ‘Unintelligible is replaced with   نشیب و فراز  The Indian Urdu replacements for ‘caterpillar’ and ‘statistician’ are utilised. | 1. These are Urdu words that retain the appropriate number of syllables. 2. This is an Urdu word suggested that retains the appropriate number of syllables.   The Indian Urdu replacements for ‘caterpillar’ and ‘statistician’ retain the same number of syllables as the original words and are well known by British Urdu speakers, so they were utilised. | | |
| **11: Language** |  | | |
| 1. The second saying is replaced with the saying that translated to ‘You cannot clap with one hand’.   The first saying, ‘All that glitters is not gold’, is translated into Urdu. | 1. The second saying, when translated into Urdu, did not retain its meaning and the saying itself was not considered well known. The saying we proposed according to the guidelines has the same meaning as ‘It takes two to tango’, and was considered a well known saying often spoken amongst British Urdu speakers.   The first saying, when translated into Urdu still retained its meaning and was considered to be a commonly spoke saying among British Urdu speakers. | | |
| **12: Language** |  | | |
| 1. Kangaroo is replaced with a sheep. 2. Penguin is replaced with a parrot or a peacock. 3. Anchor is replaced with a light bulb, hammer or scissors. 4. Harp is replaced with a dohl or a piano. 5. Rhino is replaced with a lion or a monkey. 6. Barrel is replaced with a box or suitcase. 7. Crocodile is replaced with a rabbit or zebra. 8. Accordion is replaced with a trumpet, flute or guitar.   Spoon is retained.  Book is retained.  Camel is retained.  Crown is retained. | 1. A goat was suggested due to its use in the Indian Urdu ACE-III, but they were found to be not very common in the UK. As sheep are they were proposed to replace a kangaroo, which is not common in the UK and not well known to British Urdu speakers. 2. Parrot and peacock were proposed as they are both unique birds that are better known among British Urdu speakers. 3. British Urdu speakers may struggle to identify the anchor. As an anchor has a specific purpose other objects with specific purposes were considered, narrowing down to a light bulb, hammer and scissors. 4. The harp is not a particularly familiar instrument in South Asian culture and so we proposed better known instruments such as a dohl or a piano. 5. Wild animals that were better known by British Urdu speakers were proposed. 6. British Urdu speakers may not recognise the barrel or deem it offensive due to its role as a keg containing alcohol and their views as Muslims. Other objects that act as containers were proposed. 7. A crocodile was not considered common in the UK. A lizard was suggested due to how common they are in South Asian countries but as they aren’t found in the UK it was ruled out. A rabbit or zebra were proposed, due to being better known animals. 8. An accordion may not be well recognised by British Urdu speaker. A tabla was proposed but as it is too similar to our earlier suggestion of a dohl other better known instruments were proposed.   A spoon and book are universally known across cultures.  Camels were easily recognised by participants and considered appropriate for the culture. | | |
| **13: Language** |  | | |
| Questions will be determined according to the pictures selected in Item 12. |  | | |
| **14: Language** |  | | |
| Words used in the Indian Urdu ACE-III were utilised | The words used in the Indian Urdu ACE-III were all irregular and were considered to be well known by British Urdu speakers. | | |
| **17: Visuospatial Abilities** | | |  |
| 1. K is replaced with the letter و 2. T is replaced with the letter ی   The phonetic equivalents of M and A are retained. | | 1. The letters proposed were easily recognisable by Urdu speaking elderly, having a unique shape with no dots to avoid confusion. 2. See above.   The phonetic equivalents of M and A were considered to have a unique shape that would be easily recognised. | |
| **18: Memory: Refer to Question 6: Memory** | |  | |
| **19: Memory: Refer to Question 6: Memory** | |  | |
